# Multi-year incubation experiments boost confidence in model projections of long-term soil carbon dynamics

**DOI:** 10.1038/s41467-020-19428-y

**Published:** 2020-11-17

**Authors:** Siyang Jian, Jianwei Li, Gangsheng Wang, Laurel A. Kluber, Christopher W. Schadt, Junyi Liang, Melanie A. Mayes

**Affiliations:** 1 Department of Agricultural and Environmental Sciences, Tennessee State University, Nashville, TN 37209 USA; 2 Institute for Environmental Genomics and Department of Microbiology & Plant Biology, University of Oklahoma, Norman, OK 73019 USA; 3 State Key Laboratory of Water Resources and Hydropower Engineering Sciences, Wuhan University, Wuhan, 430072 China; 4 Biosciences Division & Climate Change Science Institute, Oak Ridge National Laboratory, Oak Ridge, TN 37831 USA; 5 Environmental Division & Climate Change Science Institute, Oak Ridge National Laboratory, Oak Ridge, TN 37831 USA; 6 College of Grassland Science and Technology, China Agricultural University, Beijing, 100193 China

**Keywords:** Ecology, Biogeochemistry

## Abstract

Global soil organic carbon (SOC) stocks may decline with a warmer climate. However, model projections of changes in SOC due to climate warming depend on microbially-driven processes that are usually parameterized based on laboratory incubations. To assess how lab-scale incubation datasets inform model projections over decades, we optimized five microbially-relevant parameters in the Microbial-ENzyme Decomposition (MEND) model using 16 short-term glucose (6-day), 16 short-term cellulose (30-day) and 16 long-term cellulose (729-day) incubation datasets with soils from forests and grasslands across contrasting soil types. Our analysis identified consistently higher parameter estimates given the short-term versus long-term datasets. Implementing the short-term and long-term parameters, respectively, resulted in SOC loss (–8.2 ± 5.1% or –3.9 ± 2.8%), and minor SOC gain (1.8 ± 1.0%) in response to 5 °C warming, while only the latter is consistent with a meta-analysis of 149 field warming observations (1.6 ± 4.0%). Comparing multiple subsets of cellulose incubations (i.e., 6, 30, 90, 180, 360, 480 and 729-day) revealed comparable projections to the observed long-term SOC changes under warming only on 480- and 729-day. Integrating multi-year datasets of soil incubations (e.g., > 1.5 years) with microbial models can thus achieve more reasonable parameterization of key microbial processes and subsequently boost the accuracy and confidence of long-term SOC projections.

## Abstract (web summary)

As the climate warms, soil carbon stores will likely be degraded by microbes and released as CO_2_, but these predictions are based on laboratory incubations that might not reflect real rates. Here the authors optimize model projections using dozens of short- and long-term incubations in forest and grasslands.

## Introduction

Current Earth system models (ESMs) estimated soil carbon (C) storage that varied six-fold across eleven models in the Coupled Model Intercomparison Project Phase 5 (CMIP5)^1^ and produced highly uncertain projections about the fate of soil C in response to climate and environmental changes^2^. Coupling of select soil microbial processes into ESMs can improve soil C projections and reduce uncertainty of climate-carbon feedbacks^3^. When soil microbial models are applied at larger scales, the empirical relationships on which the models are built must be extrapolated over space and through time to predict soil C dynamics across diverse biogeochemical conditions. However, which microbial processes are important and how they are represented in the large-scale models remain challenging^4,5^. Due to the scale-dependent ecological processes, the diversity of microbial metabolic and physiological strategies, significant simplifying assumptions are proposed to link short-term soil microbial decomposition processes to decadal or longer-term projections in ESMs^4^. Further improvements are sought by explicit incorporation of microbial processes and the corresponding parameterization given rigorous model testing and validation^6^. In particular, microbial growth and maintenance are key controls of soil organic carbon (SOC) decomposition^7–9^, as well as microbial physiological traits such as dormancy^10^, acclimation^11^, and community-level interaction^12^. To simulate these microbial processes, models employ microbial parameters that are often estimated from incubation studies due to technical difficulty of in situ quantification^4,13^. In many cases, these microbial parameters are derived from short-term laboratory incubations subjected to different substrate additions at the scale of hours to several weeks^13,14^, although longer-term incubation studies exist^15–17^. Because model parameter estimates vary with length of soil incubations^18^, it is imperative to elucidate how microbial parameters derived from incubations of varying durations inform soil C dynamics and the consequences for long-term soil C projections.

Previous studies have demonstrated that key microbial parameters differ substantially between estimates derived from short- and long-term soil incubation datasets. For instance, Hagerty and colleagues reported carbon use efficiency (CUE) was ~0.72–0.74 from a week-long laboratory incubation using ^13^C-labeled glucose in forest soils^18^, while Li and colleagues reported averaged CUE was ~0.39–0.42 by assimilating 22-year-long field soil warming data^19^. As one of the most sensitive parameters to SOC simulation, these different CUE estimates may impart divergent model projections^5,11^. The differences in CUE estimates may be due to different quantification methods of the differential microbial mechanisms operating at contrasting spatiotemporal scales^13^, e.g., day vs. decade and population vs. community vs. ecosystem^20^. Also, CUE declines with warming based on laboratory studies, but increases with higher mean annual temperature (MAT) across biomes to capture the large-scale soil respiration patterns^21–24^. Furthermore, the need for integrating a constant or dynamic CUE value across biomes has not been determined because of high variation of substrate quality, enzyme kinetics, and even physical constraints such as mineral occlusion^21,25–29^.

Another example is that the maximum specific microbial growth rates (*V*_g_) were 0.17 h^−1^ and 0.12 h^−1^ using hour-long substrate-induced respiration experiments^30,31^, while *V*_g_ was estimated to be 0.01–0.05 h^−1^ using data from a 270-day soil incubation^10^. These straightforward comparisons suggest that parameters estimated from short- versus long-term datasets may differ by at least an order of magnitude. Microbial parameter estimates may be inevitably overestimated in short-term incubations because microbial uptake and growth rates may be stimulated due to higher substrate availability^32,33^. However, it remains unclear whether implementing parameter values derived from longer-term studies will result in contrasting model projections. Furthermore, it is important that such mechanistic model projections be validated with observations (e.g., a meta-analysis).

In this study, three incubation datasets derived from the same soils but of varying lengths (6 days, 30 days, and 729 days) and subjected to two different substrate additions (i.e, glucose and cellulose)^34^ were used to tune a soil microbial model and obtain best-fit microbial parameters. The 6 days incubation was subjected to glucose addition, and the 30 and 729 days incubation were subjected to cellulose addition^34^. By implementing these parameters in the Microbial-ENzyme Decomposition (MEND) model, SOC responses to warming were projected and compared with observations. Different from the first-order decay model, MEND is a typical soil microbial model characterized by dependence of soil C fluxes on microbial biomass or enzyme pools^5^. The MEND model incorporates key microbial parameters related to microbial growth and maintenance, community physiology, mortality, and dormancy^10,19,35–37^ and the multiple data types originating from the incubations also met the MEND model needs. Five key microbial parameters were derived from the incubation data including the intrinsic carbon use efficiency (*Y*_g_; closely related to CUE), initial active fraction of microbes (*r*_0_), half-saturation constant for microbial assimilation of substrate (*K*_D_), maximum specific growth rate (*V*_g_), and a ratio (*α* = *V*_mt_/(*V*_g_ + *V*_mt_)) that determines the relationship between *V*_g_ and the specific maintenance rate (*V*_mt_; see Methods section for detailed descriptions). The three sets of parameters derived from the two short- and one long-term datasets (hereafter short-term glucose parameters, ST_G_; short-term cellulose parameters, ST_C_; and long-term cellulose parameters, LT_C_) were implemented to project the SOC change in response to 5 °C warming over five decades. To validate the three sets of parameters, the model projections were compared with a meta-analysis of SOC responses to field warming manipulations of variable duration that consisted of 149 observations.

We first hypothesize that the best-fit short- and long-term parameter estimates differ significantly such that *Y*_g_, in particular, is consistently higher in the two short-term datasets than in the long-term dataset. This is due to the initially high substrate availability and consequently stimulated microbial growth and activity over the short term (i.e., hours to days). Secondly, we hypothesize that implementing the best-fit short- and long-term parameter estimates results in distinct model projections of SOC response to warming, which are primarily driven by different *Y*_g_. That is, when *Y*_g_ is derived from the short-term datasets the parameter value is inflated and it will lead to higher microbial biomass and enzyme production, faster SOC decomposition rate, and consequently exceed the SOC replenishment rate resulting in a net SOC loss; on the contrary, a net SOC loss can revert to a net gain given the smaller *Y*_g_ derived from long-term dataset. Lastly, we hypothesize that the model projections of SOC response to warming by implementing the long-term parameters are more consistent with the field observations, and the projections based on the short-term parameters overestimate SOC response to warming, and thus the use of long-term datasets to derive critical model parameters are more appropriate for projecting soil responses to climate warming that occurs over decades.

## Results

### The best-fit short-term and long-term parameters

**Fig. 1:**
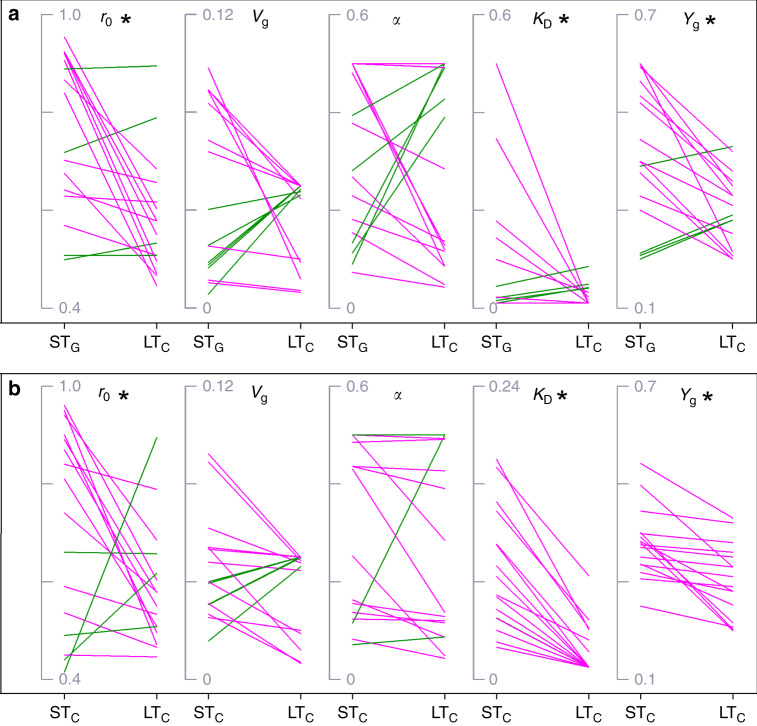
Parameter estimates based on three different datasets. Estimates of five best-fit parameters (initial active fraction, *r*_0_; maximum specific growth rate, *V*_g_; a ratio relating maintenance rate to growth rate, *α*; half-saturation constant, *K*_D_; intrinsic carbon use efficiency, *Y*_g_) derived from 16 short-term glucose (ST_G_), 16 short-term cellulose (ST_C_), and 16 long-term cellulose (LT_C_) datasets. **a**, **b** denote estimates for ST_G_ vs. LT_C_, and ST_C_ vs. LT_C_, respectively. The two ends of each line represent the parameter values derived from the short- and long-term dataset, respectively. The pink and green lines represent the declining and increasing parameter values, respectively. Asterisks denote significant differences in the parameter values derived from the short- and long-term dataset comparisons based on the Kruskal–Wallis (KW) test at a significance level of 0.05. These were consistent across both the ST_G_ vs. LT_C_ and ST_C_ vs. LT_C_ dataset comparisons.

For each of five parameters (*r*_0_, *V*_g_, *α*, *K*_D_, *Y*_g_), the 16 pairs (4 soil types × 2 ecosystems × 2 substrate treatments) of best-fit ST_G_ and LT_C_ parameter estimates were compared and 10–12 of the parameter estimates for ST_G_ were consistently higher than their long-term counterparts for LT_C_, while the rest were smaller (Fig. 1a). That is, 4 to 6 of 16 ST_G_ parameters were lower than the LT_C_ parameters though the difference between the means of these cases was not statistically significant (*P* > 0.05). Averaged across 16 cases, the ST_G_ and LT_C_ parameter values were 0.76 and 0.59 (*r*_0_), 0.048 and 0.038 (*V*_g_), 0.30 and 0.27 (*α*), 0.12 and 0.02 (*K*_D_), and 0.42 and 0.30 (*Y*_g_), respectively. For three of the five parameters (*r*_0_*, K*_D_, and *Y*_g_), the ST_G_ parameter estimates were significantly higher than LT_C_ parameter estimates based on paired *t*-test (*P* < 0.05).

Similar comparisons were made between short-term cellulose (ST_C_) and long-term cellulose (LT_C_) parameter estimates. For three of the five parameters (*r*_0_, *V*_g_, and *α*), 12–13 pairs of the short-term parameter estimates were consistently higher than their long-term counterparts. That is, 3 to 4 of 16 ST_C_ parameters were lower than the LT_C_ parameters though the difference between the means of these cases was not statistically significant (*P* > 0.05). For two other parameters (*K*_D_ and *Y*_g_), all 16 pairs of the short-term parameter estimates were higher than their long-term counterparts (Fig. 1b). Averaged across 16 cases, the ST_C_ and LT_C_ parameter values were 0.72 and 0.59 (*r*_0_), 0.046 and 0.038 (*V*_g_), 0.31 and 0.27 (*α*), 0.09 and 0.02 (*K*_D_), and 0.38 and 0.30 (*Y*_g_), respectively. The mean estimates of three parameters (*r*_0_*, K*_D_, and *Y*_g_) were significantly higher (ST_C_ > LT_C_) based on paired *t*-test (*P* < 0.05).

For all five parameters, the uncertainty (i.e., distribution) of parameter estimates differed significantly between ST_G_ and LT_C_ (Supplementary Fig. 1a), and between ST_C_ and LT_C_ based on the Kruskal–Wallis (KW) test (Supplementary Fig. 1b). The ranges of two parameter (*r*_0_ and *α*) were similar between ST_G_ (or ST_C_) and LT_C_. The best-fit parameter estimates and associated ranges for all cases are presented in Supplementary Table 1. Furthermore, the parameter estimates for multiple durations of cellulose incubation dataset are presented in Supplementary Table 2. For each dataset or three combined (ST_G_, ST_C_, and LT_C_), there were few significant correlations between parameters for substrate treatments or ecosystem type (Supplementary Table 2a). The same analysis showed no significant correlations between substrate treatments within each ecosystem type for those cases that produced lower parameter values derived from ST_G_ and ST_C_ than from LT_C_ (Supplementary Table 2b).

### Model projection and synthesized observation

**Fig. 2:**
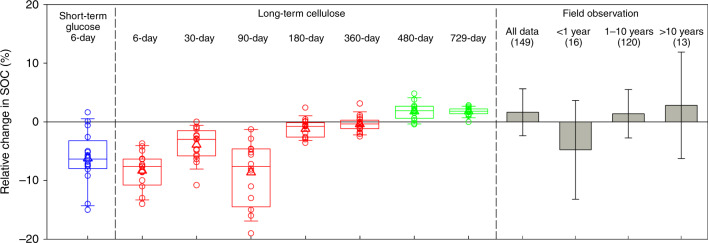
Modeled and observed soil organic carbon changes under warming. Modeled relative change (%) in soil organic carbon (SOC) based on a five-decade projection under 5 °C warming using parameters obtained from calculations using dataset of short-term glucose (6 day, blue), cellulose <1 year (6, 30, 90, 180, 360 days, red) and cellulose equal to or more than 1.5 years (480 and 729 days, green). Observations are based on the meta-analysis conducted in all field-warming experiments (all data, gray) and those over different experimental durations (e.g., <1 year, 1–10 years, and >10 years). In model results, boxplots show means (triangle), medians (line), 1st and 3rd quartiles (box, interquartile range or IQR), upper and lower extremes (whiskers). The whiskers were determined as ≤1.5 times IQR against 1st and 3rd quartiles, respectively. In model results, *N* = 16 independent runs in each box plot; In the meta-analysis, the number of observations is in parentheses.

Model projections using the short- and long-term parameters resulted in contrasting end-simulation SOC pool size and transient dynamics. By implementing the 16 sets of best-fit ST_G_, ST_C_ and LT_C_ parameters, respectively, warming (5 °C) led to a net SOC loss by 8.2 ± 5.1% (mean ± s.d.), SOC loss by 3.9 ± 2.8%, and a net SOC gain by 1.8 ± 1.0% (Fig. 2). The end-simulation SOC pool sizes under warming for all cases can be found in Supplementary Table 3. As for the transient dynamics, relative to the no warming condition, SOC declined within the first decade and then stabilized at a level of 8.2 and 3.9% loss as projected using the short-term parameters (ST_G_ and ST_C_, respectively), whereas SOC declined within the first 2 years and then gradually increased to a minor +1.8% SOC gain using the long-term parameters (LT_C_; Supplementary Fig. 2). Overall, the cellulose incubation dataset resulted in a net SOC gain of 1.7 ± 1.4% with a duration of 480 days, a net SOC loss of 0.3 ± 1.4%, 1.2 ± 1.6%, 8.6 ± 5.6%, 3.9 ± 2.8%, and 8.9 ± 3.1% with durations of 360, 180, 90, 30, and 6 days, respectively (Fig. 2).

The meta-analysis revealed that soil warming (1–5 °C) led to SOC gain by 1.6 ± 4.0% (mean ± s.d.). Over different experimental durations, warming decreased SOC stocks by 4.8 ± 8.4% for experiments lasting for <1 year, but enhanced SOC stocks by 1.4 ± 4.1% for experiments lasting for 1–10 years and 2.8 ± 9.1% in experiments lasting more than 10 years (Fig. 2). The projected changes in SOC pools at the end of the simulation are thus compared with observed SOC response to warming. Comparison showed very similar SOC changes on average with warming between simulations using the LT_C_ parameters and observations (1.8 vs. 1.6%). Comparison also showed that the simulations using the short-term parameters (ST_G_, ST_C_) departed from the observations in both sign and magnitude (–8.2%, –3.9% vs. +1.6%). Furthermore, the model projection by 480-day cellulose incubation dataset was also consistent with the observations (1.7% vs. 1.6%), whereas durations less than 480 days (i.e., 6, 30, 90, 180, and 360 days) resulted in either little change or loss of SOC (–8.9%~–0.3%; Fig. 2).

## Discussion

Results show that effects of best-fit parameter estimates depend upon experimental duration. In support of our first hypothesis, the mean parameter estimates (*r*_0_*, K*_D_, and *Y*_g_) derived from the short-term glucose and cellulose datasets were significantly higher than those derived from the long-term cellulose datasets. This reflected the microbial community dynamics that differed substantially during the short-term and long-term incubations. The relatively abundant available substrates favorable for microbial acquisition likely dominated the short-term incubations^38,39^, whereas, nutrient depletion and the consequently less available substrate would likely limit overall microbial growth and activity in the long-term incubation. In particular, it seems plausible the low microbial activities and microbial dormancy for some taxa may become more dominant over the long-term incubation experiments. However, the incubated soil samples remained relatively static over 2 years and the lack of disturbance may create artificially oligotrophic conditions that repressed microbial activities^40^. In a 22-year-long field warming experiment, soil microbial biomass and particularly fungal abundance were significantly depressed^41^, therefore, the estimates of key microbial parameters (e.g., CUE and microbial turnover) derived from the two-decade-long dataset^19^ can be up to an order of magnitude lower than those achieved based on a week-long dataset^18^. Collectively, the year-long laboratory incubation and decade-long field experiment demonstrate the advantages of assimilating long-term datasets over short-term datasets in improving microbial model parameterization.

Although the overall results supported the first hypothesis, the parameter estimates in each soil and ecosystem type individually did not consistently support it. For each parameter, 4 to 6 of 16 cases showed that the parameter estimates derived from the short-term dataset were lower than those derived from the long-term dataset, though the difference between the means was not statistically significant. Further investigation of these cases via correlation analysis showed no significant association with specific soil type, ecosystem type, or substrate type (Supplementary Table 2). Thus, these results suggest that the insignificant difference between the short- and long-term parameters may indicate an insufficient amount of useful information in each short-term case for model calibration. Indeed, the uncertainty regions of all parameter estimates were significantly different between the two short-term datasets and long-term dataset, and in particular, the uncertainty regions of *V*_g_ and *K*_D_ were much larger based on the short-term than the long-term datasets (Supplementary Fig. 1). Therefore, data of these short-term cases may have entailed larger natural variability and measurement error which was propagated through the data-model fusion procedure^42^. Thus, applying standardized measurement methods across various research sites would improve the consistency of data collection thus boosting confidence for soil modeling community^4,43^. Despite the larger number of overall observations, the low sampling frequency for certain variables (e.g., SOC and microibal biomass carbon (MBC)) in the long-term datasets may also contribute to uncertainties in parameter estimates leading to insignificant differences between short- and long-term parameter estimates.

Though glucose is more widely used as a substrate to study soil microbial responses to changing environments, the glucose incubation dataset may have a limited role in model parameterization. First, the fast and immediate response to glucose addition may allow detectable microbial responses over short term such as hours to days, since glucoses can be directly taken up by microbial community^44^. However, the diminished responses due to labile substrate depletion likely prevail over the long-term such as months to years. Cellulose addition experiments, on the contrary, would require celluloses being decomposed via enzymatic reactions to simple molecules (e.g., glucose) that could be assimilated by microbes^45^. Given the nature of the substrate and the duration of the experiment, the long-term incubation dataset would better inform microbial community dynamics compared to the short-term dataset. In fact, the fast turnover of substrate in the short-term glucose or cellulose addition experiment appears to lead to an overestimation of soil C loss in the long run. This was supported by comparing model projections and field observations in the previous paragraphs. It turns out that incubations with glucose or cellulose addition lasting <1.5 years can better project soil responses to short-term climate warming (Fig. 2). In brief, incubation datasets with cellulose addition lasting ≥1.5 years are preferred for projecting soil response to climate warming over years to decades.

**Fig. 3:**
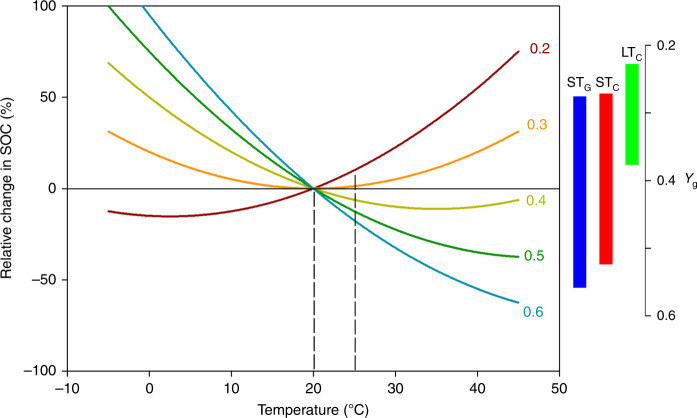
Soil organic carbon change under different temperature and *Y*_g_ (intrinsic microbial carbon use efficiency at 20 °C). Modeled relative change (%) in steady-state soil organic carbon (SOC) as a function of temperature (−10 °C to 40 °C) given a certain *Y*_g_ (e.g., 0.2, 0.3, 0.4, 0.5, and 0.6 represented by different colors). The dashed lines demonstrated the percentage change of SOC under 5 °C increase in temperature (i.e., 25 °C vs. 20 °C) under a given *Y*_g_. The bars represented the 95% confidence interval of *Y*_g_ based on short-term glucose (ST_G_, blue), short-term cellulose (ST_C_, red), and long-term cellulose (LT_C_, green) dataset. Parameters except *Y*_g_ used model default values.

Results also show that short- and long-term parameters result in contrasting model projections. In support of our second hypothesis, implementing the best-fit short- and long-term parameters resulted in contrasting projections of SOC responses to warming. On average, 8.2% (or 3.9%) SOC losses and 1.8% SOC gain were achieved based on model projections using the short- and long-term parameters, respectively. The directional differences in projections may depend on multiple mechanisms in control of the SOC pool size that rely upon multiple parameters relevant to key microbial processes such as decomposition, biomass, and physiology given the mechanistic complexity of the MEND model. However, our model sensitivity analysis showed that *Y*_g_ was the most important parameter controlling SOC change and that it has a much greater role than other parameters except *α* (Supplementary Table 4). The contrasting projections were primarily driven by different *Y*_g_. Mechanistically, warming decreased *Y*_g_ resulting in reduced microbial biomass and enzyme production^5^. Though warming could enhance maximum reaction rates for SOC decomposition and dissolved organic carbon (DOC) uptake by microbes, the depressed *Y*_g_ acted as a negative feedback on SOC decomposition and DOC uptake. Therefore, a threshold temperature value was detected at which the sign of SOC change with warming could switch^5^. As demonstrated in this study, the relative change of the steady-state SOC pool in response to temperature change was increased when 0.2 ≤ *Y*_g_ ≤ 0.32 and decreased when 0.32 < *Y*_g_ ≤ 0.6 under 5 °C of warming from 20 °C (Fig. 3). The *Y*_g_ estimate on average was 0.30, 0.38, and 0.42 derived from the long-term and short-term datasets, the three values located in two distinct ranges divided by the threshold value (e.g., 0.32), thus explaining the projected respective SOC gain and loss with the same degree of warming. This suggested that even a slight change in *Y*_g_ could lead to opposing projections of SOC change with climate warming, even given the same model and forcing data. Note that the *Y*_g_ estimate in our analyses for the long-term dataset is very similar to the global average of CUE (~0.3), whereas the *Y*_g_ estimates for the two short-term datasets are leaning toward the high-end average (~0.55–0.6) of the reported values in recent global syntheses^32,46^. Because soil CUE estimates are nearly always overestimated due to unavailable metrics for the full maintenance costs of community metabolism^32^, our results suggest that the high microbial activity during a short-term incubation may have accelerated energy spilling and deteriorated accounting maintenance costs. Therefore, such syntheses based on short-term data may have misleading effects on current model projections, and that long-term lab- and field-scale experiments may be necessary to correctly parameterize SOC models.

Results also supported our third hypothesis that the use of long-term parameters into model projections is more consistent with the field observations. That is, the long-term parameterization improved the confidence of long-term SOC projections. When using the long-term parameters (e.g., 480 and 729 days), the projected SOC gain in response to warming was very close to field observations (1.7% and 1.8% vs. 1.6%). However, the projections using the two short-term parameters (i.e., 6-day glucose and 30-day cellulose) resulted in substantial SOC losses (–8.2% and –3.9%), which were also corroborated by the varying amount of SOC loss (–8.9%~–0.3%) using the parameters of the cellulose incubation dataset with durations of 6, 90, 180, and 360 days. These projected SOC losses were overestimated as compared to the field observations over decades but were consistent with the field observations with warming for <1 year (–4.9%). As demonstrated in the previous section, our analyses show that the higher estimate of *Y*_g_ derived from the short-term datasets resulted in these substantial SOC losses under warming. Therefore, these results clearly show that parameterization derived from the model calibrations using multi-year datasets should produce more reliable projections of SOC responses to long-term climate warming. However, integration of these multiple-year or decade-long datasets into the parameterization of soil C models is still very limited because such data sets are rare in the literature. More often, the short-term soil experiments lasting hours to weeks have been used to estimate microbial parameters, which appears to lead to biased model parameterization and projections^18,47^.

Interestingly, field warming experiments lasting <1 year also seem to result in SOC loss (–4.8%) although the uncertainty is very high. This is a result similar in sign and magnitude to the SOC losses (–8.2% or –3.9%) projected using the parameters derived from our short-term glucose or cellulose datasets. Soil warming experiments often observe accelerated respiration in early stages, followed by a deceleration of respiration and a return to conditions more similar to pre-warmed rates of soil CO_2_ release^48^. The short-lived SOC losses in field experiments are often explained by depletion of labile substrate resulting from accelerated SOC decay^49–51^. This coincides with the dynamics and pattern of the soil C cycle during the short-term soil incubation experiment with the glucose amendment. Likely, this hour- to day-long microbial mechanisms identified in lab incubation somewhat captured the in situ microbial community dynamics that operated in the soil warming experiments for <1 year. That is, short-term incubation experiment may be better used to project year-long SOC response to warming, whereas, long-term incubation experiment can serve more accurately in projecting the SOC response to warming over decades. Though the short-term datasets are more common, our results clearly show that future model parameterization for long-term projections should focus on studies lasting multiple years (>1.5 years), as studies lasting <1 year often show accelerated respiration rate in warmer plots which could lead to overestimates of long-term SOC losses.

The current finding has important implications for improving soil C cycling models. In this study, the model results validated against a comprehensive data synthesis was particularly superior to former studies that only enabled different projections but lacked rigorous comparisons between simulations and observations^5^. In this data-rich era, the dramatically increased volumes of data from observational and experimental networks, along with enhanced computational power, enabled a robust data synthesis that is key to model development^6,52^. Secondly, to improve the soil microbial model calibration, it seems that more complete long-term datasets will be needed to achieve more accurate and less uncertainty in parameter estimates. As an example, soil respiration was monitored hourly but SOC, microbial biomass and exoenzyme activities were measured once every several years in the two-decade-long Harvard Forest soil experiment^19,53^. Such sampling designs resulted in powerful and detailed characterizations of soil carbon (C) loss via respiration^48^, but limited capacity in addressing the underlying microbial mechanisms governing such loss in situ. Given our finding, we advocate for new sampling designs, conducted at multiple spatiotemporal scales with more frequent (e.g. monthly or seasonally) soil collection and analysis of important variables such as SOC, microbial biomass, enzyme activities and functions, conducted at resolutions from the ecosystem level through the microbial community to genomic levels^19,54^. Thirdly, given the overall similarity in the ranges of key microbial parameters (e.g., *V*_g_ and α) for short- and long-term experiments with contrasting substrate amendments (e.g., glucose vs. cellulose) and ecosystems (e.g., forest vs. grassland), this study provided strong justifications for global constraints of key microbial parameters by assimilating the increasingly available soil data^4,6^.

In addition, the parameterization of the key microbial processes relies upon rigorous model validations against respiration and other key observations (e.g., MBC and SOC). For instance, CUE declines with warming based on laboratory studies, but a positive relationship of CUE with site level MAT was able to capture the soil respiration patterns across biomes^21–23,55^. These studies are advantageous in addressing the large-scale soil respiratory loss, but more efforts will be needed to elucidate whether projections of other key soil variables such as SOC stock are robust. On the other hand, a type of mismatch between spatiotemporal scales could occur when the site level MAT is correlated to CUE values, because these CUE estimates were generally derived from various short-term laboratory experiments, which, given our study, may be overestimated when compared with those based on long-term experiments. As a proxy of soil temperature, MAT may poorly represent the actual soil temperature and its fluctuations. Given the increasing volume of long-term experimental datasets, future studies are needed to relate the soil temperature to the increasingly available CUE derived from long-term experiments, and further validate model simulations with more variables including respiration over large scales.

Overall, this study demonstrated that the best estimates of key microbial parameters (*r*_0_, *V*_g_, *α*, *K*_D_, and *Y*_g_) differed substantially when the model calibrations were conducted using short-term datasets versus long-term datasets, with the latter calibration resulting in significantly lower parameter estimates for three parameters (*r*_0_*, K*_D_, and *Y*_g_). Implementing the best-fit short- and long-term parameters into model projections led to significant SOC loss and minor SOC gain (–8.2~–3.9% vs. 1.8%, respectively). Validated against a meta-analysis of field warming observations (1.6% ± 4.0%), this study clearly supports that the long-term parameters are more realistic to project SOC responses to warming over decades to centuries. Our model analysis elucidated that the short-term (days to weeks) datasets generated higher estimates of *Y*_g_ and the subsequent cascading effect on microbial biomass, enzymes and decomposition rates, leading to fast SOC losses and eventually overestimating long-term SOC losses with warming. Therefore, we strongly advocate for multi-year long datasets with more frequent measurements of SOC, MBC, and exoenzyme activities, which can improve the performance of microbial models and thereby achieve more reasonable parameterizations of key microbial processes. This study thus provides one of the most robust assessments of multiple large experimental and synthesized datasets to date, and corroborated the importance of long-term experiments in providing more realistic estimates of key microbial parameters for soil model improvement and future projections.

## Methods

### Incubation experiment and data collection

We collected the short- and long-term datasets from a soil incubation study conducted at Oak Ridge National Laboratory (ORNL) from 2015 to 2017^34^. The incubation experiment was introduced briefly here. In November 2014, soil samples for the incubation experiment were collected from paired forest and grassland plots in Iowa (IA), Missouri (MO), Ohio (OH), and Tennessee (TN). The description of site characteristics was provided in Supplementary Table 5. After samples were transferred to the laboratory, they were quantified for soil pH, soil total carbon content, soil total nitrogen content, dissolved organic carbon (DOC), microbial biomass carbon (MBC), particulate organic carbon (POC), and mineral-associated organic carbon (MOC) using standardized methods (Supplementary Methods). Two incubations were conducted including a short-term incubation (6 days) and a long-term incubation (729 days). In addition to the control treatment (i.e., no substrate addition), the substrate addition treatment received the equivalent of 1% of the total carbon content as ^13^C-labeled glucose or cellulose at the beginning of the short-term and long-term incubations, respectively. Though not related to the current study, the isotopically labeled substrate addition was used to for testing the carbon isotopes module in MEND^10^. Both control and substrate addition treatments were included in the study.

Soils were incubated in the dark at 22 °C throughout the incubation. Heterotrophic soil CO_2_ respiration was measured in the headspace on 7 collections in the short-term incubation (i.e., 2, 4, 8, 24, 48, 72, and 144 h) and 18 collections in the long-term incubation (i.e., 1, 2, 4, 6, 11, 20, 39, 64, 90, 120, 151, 229, 323, 390, 480, 571, 665, and 729 days). Using the chloroform fumigation-K_2_SO_4_ extraction method, soil MBC was measured on three collections in the short-term incubation (i.e., 24, 72, and 144 h) and eight collections in the long-term incubation (i.e., 1, 4, 20, 64, 151, 323, 480, and 729 days). In total, there were 48 cases (4 locations × 2 ecosystems  × 2 substrate treatments × 3 durations) and 1200 samples (16 cases × 3 replicates × 7 collections in the short-term incubation and 16 cases × 3 replicates ×  18 collections in the long-term incubation). The 16 short-term glucose (ST_G_) and 16 long-term cellulose (LT_C_) datasets were analyzed and plotted (Supplementary Figs. 3–6), and were compiled for the data-model integration in this study by aligning the time-wise measurements of the available hourly respiration and MBC. Additionally, a subset of LT_C_ dataset (e.g., the first month data) was adapted, namely short-term cellulose (ST_C_), to examine the effect of incubation duration of the same substrate on parameterization. Multiple durations of cellulose incubation dataset were also used for this analysis including 6, 90, 180, 360, and 480 days other than 30 and 729 days.

### MEND model and sensitivity analysis

Relative to a first-order decay model and other microbial models, the Microbial-ENzyme Decomposition (MEND) model is characterized by dependence of soil C fluxes on microbial and enzymatic physiology such as microbial dormancy and enzyme-catalyzed SOC decomposition^5^. The MEND model includes six categories of soil C pools (Supplementary Fig. 7): (1) particulate organic C (POC), which can be further divided into POC_1_ (denoted by *P*_1_ in Supplementary Fig. 7, containing POC degraded by oxidative enzymes) and POC_2_ (*P*_2_, containing POC degraded by hydrolytic enzymes); (2) mineral-associated organic C (MOC, *M*); (3) dissolved organic C (DOC, *D*); (4) adsorbed DOC (QOC, *Q*), which is an active layer of MOC that adsorbs and desorbs DOC; (5) active microbial biomass (*BA*) and dormant microbial biomass (BD); and (6) enzyme pools (ENZ) containing POC-degrading enzymes (EP_1_ and EP_2_ that decompose POC_1_ and POC_2_, respectively) and MOC-degrading enzymes (EM). In MEND model, the mineral-adsorbed phase of DOC (i.e., QOC) is regulated by temperature-dependent (Arrhenius) adsorption–desorption kinetics. In addition, SOC decomposition and DOC uptake follow the Michaelis-Menten equation, and the maximum reaction rate and half-saturation constant follow Arrhenius temperature dependence. External inputs in the model are separated into I_P1_, I_P2_, and I_D_ denoting inputs to the pools of POC_1_, POC_2_, and DOC, respectively. Model equations for each C pool, transformation fluxes between C pools, and model parameters were listed in Supplementary Tables 6, 7 and 8, respectively.

The MEND model was first run by setting the initial values of different C pools using the measured values in POC, MOC, DOC, and MBC pool sizes following soil sampling and prior to soil incubation (Supplementary Table 5). The initial active part of MOC (i.e., the QOC pool) was estimated as 1% of the MOC pool^35^. The initial concentrations of EP_1_, EP_2_, and EM were set to 1.1 × 10^−3^, 1.1 × 10^−3^, and 1.4 × 10^−3^ mg C cm^−3^ soil, respectively^10^. The external C input (i.e., glucose or cellulose) was 1% of the total soil C and amended on the first day of incubation in the substrate addition treatment and zero in the control treatment. The short- and long-term datasets of CO_2_ and MBC measurements were used to calibrate the MEND model.

To identify the critical microbial parameters in control of the dynamics of C pool and flux^56^, a parametric sensitivity analysis was conducted (Supplementary Table 4). The sensitivity of a C pool or CO_2_ efflux to a given parameter was assessed by the sensitivity index (*SI*)^11^:

1 $${\mathrm{SI}} = \frac{{|\log _{10}|Y_{{\mathrm{high}}}| - \log _{10}|Y_{{\mathrm{low}}}||}}{{|\log _{10}|X_{{\mathrm{high}}}| - \log _{10}|X_{{\mathrm{low}}}||}}$$

where *Y*_high_ and *Y*_low_ are the steady-state C pool or CO_2_ efflux produced by *X*_high_ (high parameter) and *X*_low_ (low parameter), which denote the high and low limit of parameter’s prior range.

### Model calibration and model projection

Based on each of the 48 datasets, a model calibration was conducted and a total of 48 model calibrations were performed. For each model calibration, five microbially relevant parameters were determined and other parameters were either set to default values or estimated from the specific soil characteristics^10^ (Supplementary Table 8). The five parameters were the initial active fraction of microbes (*r*_0_), the maximum specific growth rate (*V*_g_), the ratio (*α*) of the maximum specific maintenance rate (*V*_mt_) to (*V*_g_ + *V*_mt_), the half-saturation constant for microbial uptake of DOC (*K*_D_), and the intrinsic carbon use efficiency (*Y*_g_) at reference temperature (20 °C). The five microbial parameters were selected because they regulated microbial growth, maintenance, and transformation between dormancy and reactivation^47^. The best parameter values were determined by the modified Shuffled Complex Evolution (SCE) algorithm^10,35^, which minimized the total objective function value (*J*) that was computed as the weighted average of objective functions for CO_2_ (*J*_1_). and MBC (*J*_2_). In this study, *J*_1_ and *J*_2_ are calculated as the (1–*R*^*2*^) or the mean absolute relative error (MARE):

2 $$J = w_1 \cdot J_1 + \left( {1 - w_1} \right) \cdot J_2 = w_1 \cdot (1 - R^2) + \left( {1 - w_1} \right) \cdot {\mathrm{MARE}}$$

3 $$R^2 = 1 - \frac{{\mathop {\sum}\nolimits_{i = 1}^n {\left[ {Y_{{\mathrm{sim}}}\left( i \right) - Y_{{\mathrm{obs}}}\left( i \right)} \right]^2} }}{{\mathop {\sum}\nolimits_{i = 1}^n {\left[ {Y_{{\mathrm{obs}}}\left( i \right) - \bar Y_{{\mathrm{obs}}}} \right]^2} }}$$

4 $${\mathrm{MARE}} = \frac{1}{n}\mathop {\sum}\nolimits_{i = 1}^n {\left| {\frac{{Y_{{\mathrm{sim}}}\left( i \right) - Y_{{\mathrm{obs}}}\left( i \right)}}{{Y_{{\mathrm{obs}}}\left( i \right)}}} \right|}$$

where *w*_1_ (between 0 and 1) is the weighting factor of the objective function *J*_1_ for CO_2_ fluxes; *R*^2^ denotes the coefficient of determination (*R*^2^ ≤ 1); MARE is the mean absolute relative error (MARE ≥ 0); *n* is the number of observation; *Y*_sim_ and *Y*_obs_ were the simulated and observed values of the response variable; and $\bar Y_{{\mathrm{obs}}}$ is the mean value for *Y*_obs_.

A better model performance was evaluated by the goodness-of-fit represented by a higher *R*^2^ or lower MARE. Because *R*^2^ was not suitable for assessing the goodness-of-fit with a small set of observations^10^, MARE was used for the model calibrations against CO_2_ (*J*_1_) or MBC (*J*_2_) when the number of CO_2_ or MBC observations is <10. The satisfactory parameter estimates were obtained given MARE ≤ 0.5, a criteria applied in our previous study^10,56^. For the long-term datasets where the number of CO_2_ observation is >10, (1–*R*^2^) and MARE were used for CO_2_ (*J*_1_) and MBC (*J*_2_), respectively. The criteria for satisfactory parameter estimates were *R*^2^ ≥ 0.6 and MARE ≤ 0.5, respectively^10,56^. All model calibrations achieved the criteria of satisfactory model performance (Supplementary Table 9). The model calibrations resulted in 16 sets of best-fit ST_G_, ST_C_, and LT_C_, respectively, which were plotted and compared based on paired *t*-test. For each parameter, if the short-term parameter estimates were lower than the long-term parameter estimates, i.e., opposite to the first hypothesis, these pairs of parameters were also compared using paired *t*-test. For each dataset or three datasets combined (ST_G_, ST_C_, and LT_C_), Pearson moment correlation analysis was performed to examine whether there was a significant correlation of parameter values between substrate or ecosystem type (Supplementary Table 2). The same analysis was also conducted when the short-term parameters are smaller than long-term parameters in each dataset. These analyses were conducted using R^57^. The statistically significant test result was set at *P* < 0.05.

Furthermore, the uncertainty of short-term and long-term parameter estimates (i.e., distribution) were quantified by the Critical Objective Function Index (COFI) method^10^. The COFI was computed as *J*_cr_ (Eq. (5)). The feasible parameter space was determined by the parameters resulting in the total objective function values between *J*_opt_ and *J*_cr_. The non-parametric Kruskal–Wallis method was employed to test whether the short-term and long-term parameters significantly differed^35^. If *P* < 0.05, it indicated that the distribution of the short-term parameter estimate was significantly different from that of the long-term parameter.

5 $$J_{{\mathrm{cr}}} = J_{{\mathrm{opt}}} \cdot \left( {1 + \frac{p}{{n - p}} \cdot F_{\alpha ,\,p,\,n - p}} \right)$$

where *J*_opt_ denotes the minimum objective function value (achieved for each calibration), *n* is the number of observations, *p* is the number of calibrated parameters, and *F*_α,p,n−p_ denotes the value of the F-distribution given α = 0.05 and the degree of freedom, *p* and *n*–*p*^58,59^.

To examine the responses of soil C stocks and CO_2_ efflux under climate warming, a five-decade projection with 5 °C warming scenario was implemented using the best-fit short-term and long-term parameters given a constant external C input over time, which is not ideal but has been widely accepted and applied in other modeling studies^60,61^. This resulted in 48 model projections, 16 using the best-fit short-term glucose parameters, 16 using the best-fit short-term cellulose parameters, and 16 using the best-fit long-term cellulose parameters. The model baseline was first determined by running the model until it reached the steady state at 20 °C using the short-term and long-term parameters, respectively. Then simulations were initiated with the baseline and run for additional 50 years at 20 °C and 25 °C, respectively. The temperature-dependent slope of *Y*_g_ (*k*_Yg_) was set to −0.01^10,19^. The external C input rate was assumed to be constant over time in both 20 °C and 25 °C scenarios (i.e., annual C input  = SOC content × 5%). The climate warming effect on SOC pool size over five decades was reported as relative changes (%) of the end-simulated SOC pool size at 25 °C compared to the baseline pool size (i.e., 20 °C). The transient dynamic of SOC, MBC, and CO_2_ efflux during five-decade of warming were averaged across 16 short-term and long-term cases and plotted in Supplementary Fig. 2.

### Data synthesis of field observations

To validate the model projections, a meta-analysis was conducted based on published data of SOC pool size changes under field warming treatments. First, the dataset regarding SOC stock responses to field warming experiments was collected from two recent articles which collected publications before 2017^62,63^. Second, we searched Web of Science and extracted data from recent publications (2017–2019) using keyword combinations of warming OR increasing temperature AND soil C. Based on the two methods, a total of 149 independent observations were identified (Supplementary Table 10). The meta-analysis derived not only the effect size based on all studies but also different groups based on their experimental durations, including <1 year, 1–10 years, and >10 years. The overall and different group effect sizes were compared with the model projections using the short-term and long-term parameters, respectively. The meta-analysis was performed following the procedure as described in our previous publication^64^.

### Reporting summary

Further information on research design is available in the Nature Research Reporting Summary linked to this article.

## Supplementary information

Supplementary Information

Reporting Summary

## Data Availability

Datasets used for the modeling study is available online as in Kluber et al.^34^ and can be downloaded from https://tes-sfa.ornl.gov/sites/default/files/Soil_Respiration_Microbial_Biomass_From_Soil_Incubations_20170519.csv).

## Code availability

The MEND model code used in this study is publicly accessible at https://github.com/wanggangsheng/MEND_mult.git
