# Supplementary material for: Multi-year incubation experiments boost confidence in model projections of long-term soil carbon dynamics
Source: Nat Commun. 2020 Nov 17;11:5864. doi: 10.1038/s41467-020-19428-y (PMC7672078; doi:10.1038/s41467-020-19428-y)
Supplement: Supplementary file 1 — Supplementary Information [file 41467_2020_19428_MOESM1_ESM.pdf]

Supplementary Information for

Multi-year Incubation Experiments Boost Confidence in Model Projections of Long-term Soil Carbon Dynamics

by

Siyang Jian et al

Supplementary Information

Multi-year Incubation Experiments Boost Confidence in Model Projections of Long-term Soil Carbon Dynamics

Siyang Jian[1,2], Jianwei Li[1†], Gangsheng Wang[3†], Laurel A. Kluber[4], Christopher W. Schadt[4], Junyi Liang[5,6] and Melanie A. Mayes[5]

[1] Department of Agricultural and Environmental Sciences, Tennessee State University, Nashville, TN 37209, USA

[2] Institute for Environmental Genomics and Department of Microbiology & Plant Biology, University of Oklahoma, Norman, Oklahoma, 73019, USA

[3] State Key Laboratory of Water Resources and Hydropower Engineering Sciences, Wuhan University, Wuhan, 430072, China

[4] Biosciences Division & Climate Change Science Institute, Oak Ridge National Laboratory, Oak Ridge, Tennessee 37831, USA

[5] Environmental Division & Climate Change Science Institute, Oak Ridge National Laboratory, Oak Ridge, Tennessee 37831, USA

[6] College of Grassland Science and Technology, China Agricultural University, Beijing 100193, China

Supplementary Methods

Soil incubation experiment

Soil collection and analysis - Soils were collected in the fall of 2014 from paired forest and grassland plots within each of four different soil types in four different U.S. states, as described in ref.[1]. Sampling locations are provided in Table S4. The paired forest and grassland sites were located within 1 mile of each other and mapped to the same soil series, according to the U.S. Department of Agriculture Natural Resources Conservation Service (NRCS) soil surveys[2].

At each plot, three representative locations were identified for subsamples. Litter and plant material were removed to reveal the mineral soil and the upper 15 cm was collected with a coring device from an area of ~90 cm^2. Samples were transported on ice and stored at 4 °C. After samples were transferred to the laboratory, soil texture, soil pH, soil total carbon (C) content, soil total nitrogen (N) content, dissolved organic carbon (DOC), microbial biomass carbon (MBC), particulate organic carbon (POC) and mineral-associated organic carbon (MOC) were determined using standard methods[1]. Fresh soil samples were used for conducting these measurements except for total C and N which used air-dried soils.

The soil texture was determined by the Bouyoucos hydrometer method[3]. The soil pH was determined by shaking 1 part soil in 2 parts Milli-Q (MQ) water and measuring the pH of the supernatant[4]. The soil total C and total N contents were determined by combustion method using a Leco combustion analyzer (Leco Corp., St. Joseph, MI)[5]. The MBC was determined by the chloroform fumigation extraction method by dividing the C concentration (difference in C between fumigated and non-fumigated sub-samples) by the extraction efficiency k, which is

estimated as 0.45[6, 7]. The POC and MOC were separated by size-based fractionation and determined using Leco combustion analyzer (Leco Corp., St. Joseph, MI)[8].

Microcosm setup - The three subsamples from each of the eight plots were combined, homogenized, and sieved to 4 mm. Soil water content was adjusted to 30 % gravimetric water content (GWC) and soils were pre-incubated at 22 °C for 3 days before incubations began. Microcosms were constructed with 30 g soil in pre-weighed specimen cups, then placed in ~1 L glass mason jars for the incubation experiment. The use of specimen cups aided in sample handling during additions and monitoring water content throughout the incubations.

Microcosms received the equivalent of 1 % of the total carbon content as either ^{13}C labeled glucose (short-term) or ^{13}C labeled cellulose (long-term). To prepare the substrate additions, 99 atom percent ^{13}C labeled glucose (Sigma Aldrich, USA) and cellulose (Cambridge Scientific, USA) were diluted with unlabeled glucose and cellulose such that final additions were labeled at 15 atom %. Glucose solutions were prepared with sterile DI water such that 1 ml glucose solution was added to each microcosm. Because cellulose is insoluble, dry additions prepared by mixing dried, ground soil (same as used in microcosms) with cellulose such that final 1 g dried soil/cellulose mixture was added to each microcosm. One ml of sterile DI water was added to each microcosm after mixing the soil/cellulose addition with soils to moisten the dried soils and obtain reach the desired 30% GWC target.

Control microcosms for the short-term incubations were prepared by adding 1 ml sterile DI water, while control microcosms for the long-term incubations were prepared by adding 1 g dried ground soil and 1 ml sterile DI water. For each of soil × treatment combination, 16 replicate microcosms were prepared for short term incubations and 32 microcosms were prepared for long term incubations. In all, 768 microcosms were constructed (265 short term, and

512 long term) to allow for destructive harvesting throughout the experiments and surplus microcosms to ensure enough samples in the event of laboratory errors or accidents.

All microcosms were incubated in the dark at 22 °C and loosely capped to allow for oxygen exchange while preventing drying. Soil moisture content was monitored regularly and sterile DI water was added as needed to maintain 30% GWC.

Sampling and analysis - At each time point, three replicates of each sample type were randomly selected. Jars were flushed with room air and capped with airtight lids fitted with a septum for 2 to 24 hours prior to sampling (length of time capped was dependent on expected activity). At the time of sampling, 15 ml gas samples were taken with airtight plastic syringes and injected into pre-evacuated 12 ml Exetainer vials (Labco Limited, UK). For destructive harvesting, once gas samples were taken, jars were opened and soils were partitioned into subsamples for analysis. Approximately 5 g were oven dried at 65 °C (dry archive), ~2 g were placed in microcentrifuge tubes and stored at -80 °C to preserve for molecular analysis, ~15 g were frozen at -20 °C for MBC analysis, and the remaining soil was frozen in a separate bag and stored at -20 °C as a frozen archive.

Gas samples were submitted to EcoCore Analytical Laboratory at Colorado State University and analyzed for CO_2 concentration and isotopic signature (^{13}C-CO_2) with a modified Precon device coupled to a Thermo Fisher Delta V Advantage Isotope Ratio Mass Spectrometer (Thermo Fisher, Bremen, Germany). Soil respiration flux (*F*) was calculated as $F = \frac{\partial C}{\partial t} \frac{V}{M} \frac{P}{RT}$ where $\frac{\partial C}{\partial t}$ is the change in headspace CO_2 concentration (ppm) over the measurement period. *V* is microcosm volume, *M* is the soil dry mass, *P* is the atmospheric pressure (1 atm), *R* is the universal gas constant (82.05 mL atm mol^{-1} K^{-1}), and *T* is the air temperature (K). The final respiration rate is expressed on a dry soil basis as µg CO_2-C g^{-1} h^{-1}.

Dissolved organic carbon (DOC) and MBC were determined using the K_2SO_4 extraction and chloroform fumigation-extraction method[6, 7]. Briefly, 7 g soil was combined with 35 ml of 0.5M K_2SO_4 and placed on a shaker for 1 hour then filtered through Whatman #4 filters. A second set of soils were placed in a desiccator with 20 ml chloroform under a vacuum of 11 atm and fumigated for 48 hours prior to extraction with K_2SO_4 (as described above). Filtrate C content was determined with the combustion catalytic oxidation method on a Shimadzu TOC-L analyzer (Shimadzu Corp., Kyoto, Japan). Unfumigated samples represent DOC and MBC is calculated as the difference between fumigated and unfumigated samples.

MEND analytical steady-state solution

The major difference of the MEND steady-state solution between the version of ref.[9] and the version of ref.[10] was the introduction of microbial dormancy and the maximum microbial mortality rate ($\gamma \cdot V_{\mathrm{mt}}$) of BA and the dormant microbial pools (BD). In ref.[9] γ was assumed to be $(1 - p_{EM} - p_{EP})$, whereas in ref.[10] γ was given a range of 0.1~20. Incorporating BD and β (i.e., the ratio of dormant maintenance to V_{mt}; Table S7) change the steady-state solutions of major C pools.

First by making Eq. S7=0 (Table S5):

$$\frac{dBD}{dt} = (F_7 - F_8) - F_{11} = 0 \;\rightarrow\; BD = BA \cdot \frac{K_D}{D + \beta(D + K_D)}$$

Then making Eq. S6=0:

$$\frac{dBA}{dt} = F_6 - (F_7 - F_8) - (F_9 + F_{10}) - F_{12} - \left(F_{13,EP1} + F_{13,EP2} + F_{13,EM}\right) = 0 \rightarrow$$

$$D = \frac{K_D \cdot \left[(X \cdot V_{\mathrm{mt}} - \beta\left(V_{\mathrm{g}} - 2XV_{\mathrm{mt}}\right) + \sqrt{X^2V_{\mathrm{mt}}^2 + \beta^2(V_{\mathrm{g}} + 2V_{\mathrm{mt}})^2 + 2\beta \cdot V_{\mathrm{mt}}((2 + X)V_{\mathrm{g}} + 2V_{\mathrm{mt}})}\right]}{2(1 + \beta)(V_{\mathrm{g}} + V_{\mathrm{mt}} - X \cdot V_{\mathrm{mt}})}$$

where $X = (\gamma + p_{EM} + p_{EP})$.

β is often assumed 0.001[10]. By neglecting β, $D = K_D / \left[\frac{1}{\alpha \cdot X} - 1\right]$.

Then making Eq. S12=0:

$$\frac{d}{dt}(P_1 + P_2 + M + Q + D + BA + BD + EP_1 + EP_2 + EM)$$

$$= I_{P1} + I_{P2} + I_D - (F_9 + F_{10} + F_{11}) = 0$$

$$\rightarrow BA = \frac{\mathrm{I_D} + \mathrm{I_{P1}} + \mathrm{I_{P2}}}{\left(\frac{1}{Y_{\mathrm{g}}} - 1\right)(V_{\mathrm{g}} + V_{\mathrm{mt}}) \cdot \alpha \cdot X}$$

$$BD = \frac{\mathrm{I_D} + \mathrm{I_{P1}} + \mathrm{I_{P2}}}{\left(\frac{1}{Y_\mathrm{g}} - 1\right)(V_\mathrm{g} + V_\mathrm{mt})\frac{\alpha \cdot X(\alpha \cdot X + \beta)}{(1 - \alpha \cdot X)}} \approx \frac{\mathrm{I_D} + \mathrm{I_{P1}} + \mathrm{I_{P2}}}{\left(\frac{1}{Y_\mathrm{g}} - 1\right)(V_\mathrm{g} + V_\mathrm{mt})\frac{(\alpha \cdot X)^2}{(1 - \alpha \cdot X)}}$$

By making Eqs. S8, S9, S10=0, we can derive:

$$EP_1 = \frac{P_1 \cdot p_{EP} \cdot V_\mathrm{mt} \cdot BA}{(P_1 + P_2) \cdot r_{EP}}, EP_2 = \frac{P_2 \cdot p_{EP} \cdot V_\mathrm{mt} \cdot BA}{(P_1 + P_2) \cdot r_{EP}}, EM = \frac{p_{EM} \cdot V_\mathrm{mt} \cdot \mathrm{BA}}{r_{EM}}$$

Finally, making Eqs. S1+S2=0, S3=0 and S4=0 we derive:

$$P_1 + P_2 = POC$$

$$= \frac{\mathrm{K}_P}{\frac{P_{EP} \cdot V_P \cdot Y_g}{r_{EP}} \cdot \frac{\left(\frac{I_D}{I_P} + 1\right)}{X \cdot (1 - Y_\mathrm{g}) + Y_\mathrm{g} \cdot \gamma \cdot (1 - g_D) \cdot \left(\frac{I_D}{I_P} + 1\right)} - 1}$$

with the assumption of $V_P = V_{P1} = V_{P2}$ and $K_P = K_{P1} = K_{P2}$.

$$M = \frac{\mathrm{K}_M}{\frac{P_{EM} \cdot V_M \cdot Y_g}{r_{EM} \cdot (1 - f_D)} \cdot \frac{\left(\frac{I_D}{I_P} + 1\right)}{X \cdot (1 - Y_\mathrm{g}) + Y_\mathrm{g} \cdot \gamma \cdot (1 - g_D) \cdot \left(\frac{I_D}{I_P} + 1\right)} - 1}$$

$$Q = \frac{Q_\mathrm{max}}{1 + \frac{1}{D \cdot k_{BA}}}$$

Observed soil warming responses based on a meta-analysis

We compiled data on soil carbon responses to warming as observed field warming experiments. We used the Web of Science™ (Thomson Reuters, New York, NY) and Google Scholar (Google Inc., Mountain View, CA) to search the literature. We used the terms: (warming OR eleva* temperature) + soil carbon + field. Means and sample sizes had to be reported. For each site we collected ancillary information, e.g. latitude, longitude, warming method, average temperature increase, and biome (tundra, grassland, shrubland, forest, and desert), climate and soil properties. Common methods to elevate temperatures in the field included the use of open-top chambers (OTCs), heating cables and infrared (IR) heaters.

Soil carbon stocks were converted to kg $C \cdot m^{-2}$. If soil C was reported assoil organic matter (SOM, %), we multiplied SOM by 0.45 (assuming that 45% of SOM is C)[11]. To convert soil C data from a volume or weight basis to an area basis we used the bulk density of the soil[11].

We weighted the observations based on the duration of the study and replication as follows: $w = (n_c \times n_w)/(n_c + n_w) + (year_c \times year_w)/(year_c + year_w)$[12], where n_c and n_w representing the number of replicates and $year_c$ and $year_w$ representing the average duration over which the soil carbon data was collected in control and warmed sites, respectively. This weighting scheme assigned higher weights to well-replicated, long-term studies, as results from these studies should be the most reliable[11]. Due to standard deviations were missing for several observations, this weighting scheme is preferred in our meta-analysis over the more conventional inverse of the mixed-model variance (i.e. observations with small variance receive heavier weights)[11]. The dataset used for meta-analysis is presented in Table S9.

Supplementary Tables

Supplementary Table 1. Five best-fit microbial parameter values (r_0, V_g, α, K_D, Y_g) and their 90% percentiles based on model calibrations using the short-term glucose (ST_G), short-term cellulose (ST_C), and long-term cellulose (LT_C) datasets. ST_G/LT_C and ST_C/LT_C denote the ratio of the best-fit values derived from short-term glucose over long-term cellulose datasets, and those derived from the short-term cellulose over long-term cellulose datasets, respectively. r_0: the initial active fraction of microbes; V_g: the maximum specific growth rate; α: the ratio of the maximum specific maintenance rate (V_{mt}) to (V_g+V_{mt}); K_D: the half-saturation constant for microbial uptake of DOC; and Y_g: the intrinsic carbon use efficiency at reference temperature (20 °C).

(a) r_0			ST_G		ST_C		LT_C		ST_G/LT_C	ST_C/LT_C
Iowa	Forest	Control	0.70	*(0.41, 0.89)*	0.66	*(0.01, 0.78)*	0.66	*(0.08, 0.85)*	107%	100%
		C added	0.72	*(0.52, 0.79)*	0.84	*(0.43, 0.91)*	0.79	*(0.31, 0.98)*	91%	106%
	Grassland	Control	0.50	*(0.21, 0.74)*	0.59	*(0.01, 0.67)*	0.53	*(0.42, 0.68)*	94%	111%
		C added	0.92	*(0.79, 1)*	0.74	*(0.29, 0.91)*	0.58	*(0.15, 0.73)*	160%	128%
Missouri	Forest	Control	0.87	*(0.73, 0.97)*	0.94	*(0.47, 0.99)*	0.69	*(0.24, 0.85)*	126%	137%
		C added	0.95	*(0.81, 1)*	0.96	*(0.38, 0.99)*	0.60	*(0.2, 0.84)*	158%	159%
	Grassland	Control	0.64	*(0.11, 0.89)*	0.89	*(0.20, 0.99)*	0.58	*(0.01, 0.73)*	111%	154%
		C added	0.92	*(0.56, 1)*	0.90	*(0.23, 0.99)*	0.50	*(0.11, 0.62)*	186%	181%
Ohio	Forest	Control	0.92	*(0.69, 1)*	0.87	*(0.34, 0.99)*	0.55	*(0.31, 0.66)*	168%	158%
		C added	0.57	*(0.01, 0.93)*	0.81	*(0.19, 0.99)*	0.51	*(0.01, 0.79)*	112%	159%
	Grassland	Control	0.68	*(0.4, .92)*	0.54	*(0.01, 0.74)*	0.47	*(0.01, 0.57)*	145%	115%
		C added	0.91	*(0.71, 1)*	0.95	*(0.39, 0.99)*	0.47	*(0.01, 0.61)*	192%	202%
Tennessee	Forest	Control	0.63	*(0.05, 0.94)*	0.44	*(0.11, 0.72)*	0.62	*(0.54, 1)*	102%	71%
		C added	0.89	*(0.59, 1)*	0.42	*(0.11, 0.69)*	0.90	*(0.42, 1)*	99%	46%
	Grassland	Control	0.51	*(0.04, 0.71)*	0.49	*(0.01, 0.59)*	0.51	*(0.18, 0.69)*	100%	96%
		C added	0.84	*(0.42, 0.93)*	0.45	*(0.08, 0.60)*	0.45	*(0.31, 0.79)*	188%	101%

(b) V_g			ST_G		ST_C		LT_C		ST_G/LT_C	ST_C/LT_C
Iowa	Forest	Control	0.02	*(0.02, 0.07)*	0.04	*(0.02, 0.05)*	0.05	*(0.01, 0.05)*	33%	80%
		C added	0.02	*(0.01, 0.02)*	0.09	*(0.04, 0.1)*	0.05	*(0.01, 0.05)*	35%	185%
	Grassland	Control	0.09	*(0.01, 0.1)*	0.05	*(0.01, 0.05)*	0.05	*(0.01, 0.05)*	178%	107%
		C added	0.04	*(0.01, 0.06)*	0.06	*(0.03, 0.06)*	0.05	*(0.01, 0.05)*	84%	130%
Missouri	Forest	Control	0.09	*(0.01, 0.09)*	0.05	*(0.01, 0.05)*	0.04	*(0.01, 0.05)*	200%	107%
		C added	0.03	*(0.003, 0.05)*	0.02	*(0.01, 0.03)*	0.05	*(0.01, 0.05)*	56%	34%
	Grassland	Control	0.06	*(0.01, 0.1)*	0.03	*(0.01, 0.04)*	0.05	*(0.05, 0.05)*	128%	61%
		C added	0.02	*(0.01, 0.07)*	0.04	*(0.01, 0.05)*	0.05	*(0.05, 0.05)*	37%	79%
Ohio	Forest	Control	0.08	*(0.01, 0.09)*	0.03	*(0.02, 0.05)*	0.05	*(0.03, 0.05)*	168%	61%
		C added	0.01	*(0.003, 0.02)*	0.09	*(0.03, 1)*	0.05	*(0.02, 0.05)*	12%	183%
	Grassland	Control	0.10	*(0.02, 0.1)*	0.05	*(0.02, 0.05)*	0.01	*(0.01, 0.04)*	822%	451%
		C added	0.01	*(0.004, 0.05)*	0.03	*(0.01, 0.03)*	0.01	*(0.01, 0.05)*	159%	475%
Tennessee	Forest	Control	0.07	*(0.04, 0.1)*	0.05	*(0.02, 0.05)*	0.05	*(0.02, 0.05)*	137%	108%
		C added	0.01	*(0.002, 0.07)*	0.03	*(0.02, 0.04)*	0.01	*(0.01, 0.04)*	160%	375%
	Grassland	Control	0.09	*(0.06, 0.1)*	0.04	*(0.01, 0.05)*	0.02	*(0.02, 0.04)*	472%	213%
		C added	0.03	*(0.05, 0.1)*	0.03	*(0.02, 0.05)*	0.02	*(0.02, 0.05)*	126%	125%

(c) α			ST_G		ST_C		LT_C		ST_G/LT_C	ST_C/LT_C
Iowa	Forest	Control	0.48	*(0.06, 0.41)*	0.15	*(0.15, 0.43)*	0.13	*(0.13, 0.35)*	374%	121%
		C added	0.50	*(0.35, 0.5)*	0.12	*(0.12, 0.5)*	0.12	*(0.12, 0.33)*	415%	103%
	Grassland	Control	0.18	*(0.08, 0.5)*	0.14	*(0.14, 0.5)*	0.12	*(0.12, 0.33)*	157%	118%
		C added	0.27	*(0.06, 0.5)*	0.16	*(0.13, 0.5)*	0.09	*(0.09, 0.27)*	312%	189%
Missouri	Forest	Control	0.15	*(0.02, 0.5)*	0.25	*(0.21, 0.5)*	0.05	*(0.05, 0.19)*	319%	524%
		C added	0.07	*(0.04, 0.49)*	0.08	*(0.05, 0.48)*	0.04	*(0.05, 0.16)*	169%	190%
	Grassland	Control	0.09	*(0.07, 0.5)*	0.12	*(0.11, 0.5)*	0.50	*(0.49, 0.5)*	18%	23%
		C added	0.13	*(0.04, 0.5)*	0.48	*(0.22, 0.5)*	0.49	*(0.47, 0.5)*	27%	99%
Ohio	Forest	Control	0.23	*(0.03, 0.5)*	0.43	*(0.19, 0.5)*	0.14	*(0.14, 0.26)*	168%	315%
		C added	0.50	*(0.06, 0.5)*	0.07	*(0.02, 0.38)*	0.09	*(0.09, 0.21)*	575%	82%
	Grassland	Control	0.11	*(0.11, 0.36)*	0.44	*(0.12, 0.5)*	0.39	*(0.28, 0.5)*	29%	112%
		C added	0.28	*(0.08, 0.5)*	0.44	*(0.12, 0.5)*	0.43	*(0.12, 0.43)*	66%	102%
Tennessee	Forest	Control	0.38	*(0.3, 0.49)*	0.50	*(0.29, 0.5)*	0.28	*(0.26, 0.4)*	133%	176%
		C added	0.50	*(0.21, 0.5)*	0.50	*(0.39, 0.5)*	0.49	*(0.2, 0.5)*	102%	102%
	Grassland	Control	0.39	*(0.37, 0.5)*	0.50	*(0.27, 0.5)*	0.50	*(0.35, 0.5)*	79%	100%
		C added	0.50	*(0.38, 0.5)*	0.50	*(0.25, 0.5)*	0.50	*(0.29, 0.5)*	100%	100%

(d) K_D			ST_G		ST_C		LT_C		ST_G/LT_C	ST_C/LT_C
Iowa	Forest	Control	0.35	*(0.17, 0.5)*	0.11	*(0.01, 0.38)*	0.01	*(0.01, 0.02)*	3464%	1100%
		C added	0.50	*(0.32, 0.5)*	0.06	*(0.02, 0.25)*	0.01	*(0.01, 0.02)*	4999%	571%
	Grassland	Control	0.01	*(0.01, 0.5)*	0.05	*(0.01, 0.36)*	0.01	*(0.01, 0.02)*	105%	502%
		C added	0.01	*(0.01, 0.5)*	0.03	*(0.03, 0.22)*	0.01	*(0.01, 0.02)*	100%	263%
Missouri	Forest	Control	0.14	*(0.02, 0.5)*	0.07	*(0.03, 0.45)*	0.01	*(0.01, 0.03)*	1432%	675%
		C added	0.01	*(0.01, 0.5)*	0.09	*(0.03, 0.41)*	0.01	*(0.01, 0.03)*	100%	928%
	Grassland	Control	0.01	*(0.01, 0.5)*	0.15	*(0.02, 0.36)*	0.04	*(0.04, 0.09)*	24%	347%
		C added	0.02	*(0.02, 0.5)*	0.14	*(0.02, 0.33)*	0.05	*(0.05, 0.15)*	43%	282%
Ohio	Forest	Control	0.02	*(0.02, 0.5)*	0.08	*(0.01, 0.44)*	0.01	*(0.01, 0.02)*	223%	840%
		C added	0.50	*(0.01, 0.5)*	0.04	*(0.04, 0.4)*	0.01	*(0.01, 0.02)*	4997%	403%
	Grassland	Control	0.18	*(0.02, 0.14)*	0.11	*(0.02, 0.37)*	0.02	*(0.02, 0.04)*	781%	485%
		C added	0.01	*(0.01, 0.5)*	0.03	*(0.01, 0.41)*	0.01	*(0.01, 0.03)*	100%	305%
Tennessee	Forest	Control	0.04	*(0.07, 0.41)*	0.17	*(0.07, 0.33)*	0.08	*(0.03, 0.09)*	52%	204%
		C added	0.01	*(0.01, 0.5)*	0.05	*(0.01, 0.42)*	0.01	*(0.01, 0.06)*	100%	500%
	Grassland	Control	0.10	*(0.04, 0.33)*	0.07	*(0.02, 0.5)*	0.03	*(0.03, 0.05)*	310%	215%
		C added	0.02	*(0.18, 0.32)*	0.18	*(0.05, 0.39)*	0.04	*(0.01, 0.06)*	38%	442%

(e) Y_g			ST_G		ST_C		LT_C		ST_G/LT_C	ST_C/LT_C
Iowa	Forest	Control	0.44	*(0.26, 0.44)*	0.50	*(0.38, 0.6)*	0.33	*(0.24, 0.54)*	135%	151%
		C added	0.60	*(0.41, 0.6)*	0.37	*(0.25, 0.48)*	0.35	*(0.24, 0.54)*	171%	106%
	Grassland	Control	0.39	*(0.2, 0.49)*	0.54	*(0.36, 0.53)*	0.43	*(0.24, 0.54)*	91%	126%
		C added	0.53	*(0.2, 0.56)*	0.40	*(0.29, 0.5)*	0.38	*(0.25, 0.56)*	140%	105%
Missouri	Forest	Control	0.21	*(0.2, 0.42)*	0.31	*(0.2, 0.47)*	0.29	*(0.24, 0.6)*	73%	106%
		C added	0.40	*(0.2, 0.41)*	0.33	*(0.2, 0.45)*	0.31	*(0.22, 0.58)*	129%	108%
	Grassland	Control	0.52	*(0.2, 0.48)*	0.38	*(0.2, 0.48)*	0.36	*(0.27, 0.42)*	144%	104%
		C added	0.21	*(0.2, 0.3)*	0.32	*(0.2, 0.34)*	0.28	*(0.25, 0.36)*	74%	114%
Ohio	Forest	Control	0.56	*(0.2, 0.54)*	0.35	*(0.2, 0.44)*	0.33	*(0.2, 0.32)*	171%	106%
		C added	0.20	*(0.2, 0.39)*	0.38	*(0.25, 0.45)*	0.28	*(0.21, 0.4)*	72%	136%
	Grassland	Control	0.40	*(0.2, 0.51)*	0.25	*(0.2, 0.34)*	0.21	*(0.2, 0.31)*	193%	121%
		C added	0.38	*(0.2, 0.59)*	0.38	*(0.2, 0.44)*	0.20	*(0.2, 0.45)*	188%	190%
Tennessee	Forest	Control	0.60	*(0.26, 0.45)*	0.39	*(0.3, 0.42)*	0.22	*(0.21, 0.41)*	278%	182%
		C added	0.59	*(0.25, 0.59)*	0.44	*(0.32, 0.48)*	0.42	*(0.2, 0.6)*	141%	106%
	Grassland	Control	0.33	*(0.25, 0.53)*	0.34	*(0.14, 0.5)*	0.25	*(0.22, 0.28)*	130%	133%
		C added	0.30	*(0.28, 0.32)*	0.40	*(0.18, 0.54)*	0.20	*(0.2, 0.57)*	148%	198%

Supplementary Table 2. Pearson-moment correlation coefficients between (a) parameter estimates in different ecosystem (forest vs. grassland) and substrate treatment (control vs. substrate addition) calculated for short-term glucose (ST_G) dataset, short-term cellulose (ST_C) dataset and long-term cellulose (LT_C) datasets individually and all three datasets combined; (b) parameter estimates in different substrate treatment within forest and grassland ecosystem. Each dataset is composed of 16 independent incubations (2 ecosystem × 2 substrate treatment × 4 location). *, **, and *** denote P<0.05, P<0.01, and P<0.001, respectively. r_0: the initial active fraction of microbes; V_g: the maximum specific growth rate; α: the ratio of the maximum specific maintenance rate (V_{mt}) to (V_g+V_{mt}); K_D: the half-saturation constant for microbial uptake of DOC; and Y_g: the intrinsic carbon use efficiency at reference temperature (20 °C).

(a)

Correlation coefficient (r)		r_0	V_g	α	K_D	Y_g
Between ecosystem	ST_G	0.17	0.63	0.68	0.5	0.15
	ST_C	0.78*	0.14	0.44	0.14	0.75*
	LT_C	0.17	0.17	0.24	0.1	0.004
	3 datasets	0.56**	0.38	0.24	0.23	0.21
Between Substrate	ST_G	0.48	0.17	0.82*	0.41	0.02
	ST_C	0.7	0.5	0.42	0.01	0.21
	LT_C	0.65	0.65	0.93**	0.24	0.34
	3 datasets	0.45*	0.23	0.69***	0.45	0.25

(b)

Correlation coefficient (r)		r_0	V_g	α	K_D	Y_g
Between treatment (forest)	3 datasets	0.49	0.46	0.64	0.48	0.21
Between treatment (grassland)	3 datasets	0.48	0.12	0.28	0.29	0.29

Supplementary Table 3. The equilibrium and end-simulation soil organic carbon (SOC) pool sizes under 5ºC warming using the short-term glucose (ST_G), short-term cellulose (ST_C) and long-term cellulose (LT_C) datasets derived parameters over 50 years (see detail in Methods section). SOC_{eq}: SOC pool size at the equilibrium state.

Cases			SOC (mg C g^{-1} soil)			
			SOCeq	ST_G	ST_C	LT_C
Iowa	Forest	Control	37.3	34.5	35.5	37.9
		C added	39.7	39.7	39.3	40.7
	Grassland	Control	33.4	28.7	32.6	34.1
		C added	36.1	34.0	35.2	36.5
Missouri	Forest	Control	48.2	44.3	45.9	49.1
		C added	49.5	45.9	48.0	50.4
	Grassland	Control	23.6	23.5	22.2	23.9
		C added	27.2	25.4	26.9	28.0
Ohio	Forest	Control	39.2	33.3	38.1	39.6
		C added	41.8	38.0	37.3	41.8
	Grassland	Control	23.8	22.6	23.1	24.4
		C added	24.1	22.3	22.7	24.6
Tennessee	Forest	Control	54.2	51.5	50.5	55.1
		C added	53.9	52.5	50.5	55.0
	Grassland	Control	31.6	32.1	31.3	32.1
		C added	33.9	32.0	33.9	34.5

Supplementary Table 4. Sensitivity index calculated for target parameters. SOC, soil organic carbon; DOC, dissolved organic carbon; MBC, total microbial biomass carbon; MBA, active microbial biomass carbon; MBD, dormant microbial biomass carbon; ENZ, enzyme pool includes EP_1, EP_2, and EM; CO_2, microbial CO_2 flux. Sensitivity index (*SI*) are calculated according to ref.[13].

Parameter	Range	Sensitivity index (*SI*)						
		SOC	DOC	MBC	MBA	MBD	ENZ	CO_2
r_0	(0.01,1)	0.00	0.00	2.55	2.18	1.90	2.18	0.00
V_g	(0.001,0.1)	0.06	0.02	7.78	18.73	17.79	17.73	0.04
α	(0.01,0.5)	1.55	0.73	7.69	16.51	16.97	15.33	0.47
K_D	(0.01,0.5)	0.29	0.86	0.75	1.19	1.35	1.19	0.05
Y_g	(0.2,0.6)	1.57	0.01	0.02	0.05	0.01	0.05	0.10

Supplementary Table 5. Sampling locations, taxonomy of soils, pH, soil carbon content, soil nitrogen content, dissolved organic carbon (DOC), microbial biomass carbon (MBC), particulate organic carbon (POC), and mineral-associated organic carbon (MOC) of soils used in the incubation study. Data in parenthesis are the standard deviations from 4 analytical replicates.

State	Ecosystem	Location	Soil Series	Soil Taxa	pH	Carbon (%)	Nitrogen (%)	DOC[a]	MBC[a]	POC (%)	MOC (%)
IA	Forest	41.79° N, 93.43° W	Coland	Cumulic Endoaquolls	6.56	3.17 (0.29)	0.27 (0.01)	50.83 (3.2)	221.00 (11.0)	1.60	98.38
	Grass				6.45	3.13 (0.62)	0.25 (0.01)	44.11 (2.8)	455.80 (17.5)	2.44	97.60
MO	Forest	38.74° N, 92.19° W	Kesswick	Aquertic Chromic Hapludalfs	5.89	4.42 (0.62)	0.22 (0.13)	76.95 (1.7)	464.04 (22.1)	4.66	95.69
	Grass				5.57	2.21 (0.08)	0.20 (0.01)	67.91 (3.2)	187.87 (11.4)	0.69	99.51
OH	Forest	39.32° N, 82.12° W	Westmoreland	Ultic Hapludalfs	5.5	4.14 (0.06)	0.31 (0.01)	271.45 (52.7)	380.54 (245.5)	3.16	97.16
	Grass				6.36	2.18 (0.08)	0.23 (0.01)	66.51 (14.3)	623.56 (45.4)	1.20	98.74
TN	Forest	35.93° N, 84.31° W	Etowah	Typic Paleudults	7.56	5.71 (0.22)	0.47 (0.07)	176.95 (54.3)	765.79 (77.3)	3.66	96.72
	Grass				7.29	3.08 (0.16)	0.33 (0.03)	96.28 (9.2)	517.88 (142.3)	0.58	99.41

[a] unit of DOC and MBC is mg C·kg^{-1} soil

Supplementary Table 6. The dynamics of each soil carbon pool in MEND model.

Carbon pool variation	Equation	
Particulate organic carbon (POC) pool 1 (P_1)	$\frac{dP_1}{dt} = I_{P1} + (1 - g_D) \cdot F_{12} - F_1$	(S1)
POC pool 2 (P_2)	$\frac{dP_2}{dt} = I_{P2} - F_2$	(S2)
Mineral-associated organic carbon (MOC,M)	$\frac{dM}{dt} = (1 - f_D) \cdot (F_1 + F_2) - F_3$	(S3)
Adsorbed DOC (QOC,Q)	$\frac{dQ}{dt} = F_4 - F_5$	(S4)
Dissolved organic carbon (DOC,D)	$\frac{dD}{dt} = I_D + f_D \cdot (F_1 + F_2) + g_D \cdot F_{12} + F_3 + \left(F_{14,EP1} + F_{14,EP2} + F_{14,EM}\right) - F_6 - (F_4 - F_5)$	(S5)
MBA	$\frac{dBA}{dt} = F_6 - (F_7 - F_8) - (F_9 + F_{10}) - F_{12} - \left(F_{13,EP1} + F_{13,EP2} + F_{13,EM}\right)$	(S6)
MBD	$\frac{dBD}{dt} = (F_7 - F_8) - F_{11}$	(S7)
Enzymes for P_1 (EP_1)	$\frac{dEP_1}{dt} = F_{13,EP1} - F_{14,EP1}$	(S8)
Enzymes for P_2 (EP_2)	$\frac{dEP_2}{dt} = F_{13,EP2} - F_{14,EP2}$	(S9)
Enzymes for M (EM)	$\frac{dEM}{dt} = F_{13,EM} - F_{14,EM}$	(S10)

Respiration (CO_2)	$$\frac{dCO_2}{dt} = (F_9 + F_{10}) + F_{11}$$	(S11)
Carbon balance	$$\frac{d}{dt}(P_1 + P_2 + M + Q + D + BA + BD + EP_1 + EP_2 + EM) = I_{P1} + I_{P2} + I_D - (F_9 + F_{10} + F_{11})$$	(S12)

Supplementary Table 7. Component fluxes in the MEND model

Flux description	Equation	
Particulate organic carbon (POC) pool 1 (P_1) decomposition (F_1)	$F_1 = V_{P1} \cdot EP_1 \cdot P_1/(K_{P1} + P_1)$	(S13)
POC pool 2 (P_2) decomposition	$F_2 = V_{P2} \cdot EP_2 \cdot P_2/(K_{P2} + P_2)$	(S14)
Mineral-associated organic carbon (MOC, *M*) decomposition	$F_3 = V_M \cdot EM \cdot M/(K_M + M)$	(S15)
Adsorption (F_4) and desorption (F_5) between dissolved organic carbon (DOC, *D*) and adsorbed DOC (QOC, *Q*)	$F_4 = k_{\mathrm{ads}} \cdot (1 - Q/Q_{\mathrm{max}}) \cdot D$<br>$F_5 = k_{\mathrm{des}} \cdot (Q/Q_{\mathrm{max}})$	(S16)<br>(S17)
DOC (*D*) uptake by microbes	$F_6 = \frac{1}{Y_{\mathrm{g}}} \cdot (V_{\mathrm{g}} + V_{\mathrm{mt}}) \frac{D \cdot BA}{K_D + D}$	(S18)
Dormancy (F_7) and reactivation (F_8) between active (MBA) and dormant (MBD) microbial biomass (*BA* and *BD*)	$F_7 = [1 - D/(K_D + D)] \cdot V_{\mathrm{mt}} \cdot BA$<br>$F_8 = D/(K_D + D) \cdot V_{\mathrm{mt}} \cdot BD$	(S19)<br>(S20)
MBA (*BA*) growth respiration (F_9) and maintenance respiration (F_{10})	$F_9 = (\frac{1}{Y_{\mathrm{g}}} - 1) \frac{V_{\mathrm{g}} \cdot D \cdot BA}{K_D + D}$<br>$F_{10} = (\frac{1}{Y_{\mathrm{g}}} - 1) \frac{V_{\mathrm{mt}} \cdot D \cdot BA}{K_D + D}$	(S21)<br>(S22)
MBD (*BD*) maintenance respiration	$F_{11} = \beta \cdot V_{\mathrm{mt}} \cdot BD$	(S23)
MBA (*BA*) mortality	$F_{12} = \gamma \cdot V_{\mathrm{mt}} \cdot BA$	(S24)

Synthesis of enzymes for P_1 (EP_1, $F_{13,\mathrm{EP1}}$), enzymes for P_2 (EP_2, $F_{13,\mathrm{EP2}}$), and enzymes for M (EM, , $F_{13,\mathrm{EM}}$)	$F_{13,EP1} = P_1/(P_1 + P_2) \cdot P_{EP} \cdot V_{\mathrm{mt}} \cdot BA$<br>$F_{13,EP2} = P_2/(P_1 + P_2) \cdot P_{EP} \cdot V_{\mathrm{mt}} \cdot BA$<br>$F_{13,EM} = P_{EM} \cdot V_{\mathrm{mt}} \cdot BA$	(S25)
Turnover of enzymes (EP_1, EP_2, EM)	$F_{14,EP1} = r_E \cdot EP_1$<br>$F_{14,EP2} = r_E \cdot EP_2$<br>$F_{14,EM} = r_E \cdot EM$	(S26)

Supplementary Table 8. Default parameter values adopted by the MEND model.

ID	Parameter	Description	Prior range	Predetermined value	Unit
1	LF_0	Initial fraction of POC_1 in POC	(0.1, 1.0)	0.3	—
2	r_0	Initial active fraction of microbes	(0.01, 1)	Calibrated	—
3	V_{P1}	Max specific decomposition rate for POC_1	(0.1, 100)	20	mg C·mg^{-1} C·h^{-1}
4	V_{P2}	Max specific decomposition rate for POC_2	(0.1, 100)	20	mg C·mg^{-1} C·h^{-1}
5	V_M	Max specific decomposition rate for MOC	(0.1, 100)	20	mg C·mg^{-1} C·h^{-1}
6	K_{P1}	Half-saturation constant (*K*) for POC_1 decomposition	(40, 100)	K_{P1} = 41.58 * [DOC] + 44.95	mg C·g^{-1} soil
7	K_{P2}	*K* for POC_2 decomposition	(1, 40)	6	mg C·g^{-1} soil
8	K_M	*K* for MOC decomposition	(100, 1000)	K_M = 455.11 * [DOC] + 400.42	mg C·g^{-1} soil
9	$Q_{\max}$	Max sorption capacity	(0.5, 5)	$Q_{\max}$ = 0.012 * [MOC] + 3.43	mg C·g^{-1} soil
10	K_{ba}	Binding affinity	(1, 16)	K_{ba} = -0.082 * (%sand) + 11.23	(mg C·g^{-1} soil)$^{-1}$
11	k_{des}	Desorption rate	(0.0001, 0.01)	k_{des} = -0.059 * [DOC] + 0.0059	mg C·g^{-1} soil·h^{-1}
12	r_E	Turnover rate of enzymes	(0.0001, 0.01)	0.003	mg C·mg^{-1} C·h^{-1}
13	p_{EP}	[$V_m \times p_{EP}$] is the production rate of *EP* (EP_1 + EP_2), V_m is the specific maintenance rate for active microbes	(0.0001, 0.05)	0.1	—
14	fp_{EM}	fp_{EM} = p_{EM}/p_{EP} , [$V_{mt} \times p_{EM}$] is the production rate of *EM*	(0.5, 3.0)	1	—
15	f_D	Fraction of decomposed POC allocated to DOC	(0.05, 1)	0.5	—
16	g_D	Fraction of dead microbes allocated to DOC	(0.01, 1)	0.5	—
17	V_g	Max specific growth rate	(0.001, 0.1)	Calibrated	mg C·mg^{-1} C·h^{-1}
18	α	= V_{mt} / (V_g + V_{mt})	(0.01, 0.5)	Calibrated	—
19	K_D	*K* for microbial uptake	(0.01, 0.5)	Calibrated	mg C·g^{-1} soil
20	Y_g	True growth yield at reference temperature (T_{ref})	(0.2, 0.6)	Calibrated	—

21	k_{Yg}	Temperature slope for Y_g	(0.001,0.016)	0.01	$(°C)^{-1}$
22	γ	Max microbial mortality rate = $V_m \times \gamma$	(0.1, 20)	1	—
23	β	Ratio of dormant maintenance rate to V_m	(0.0005,0.05)	0.001	—
24	ψ_{A2D}	Soil water potential (SWP) threshold for microbial dormancy	(−0.6, −0.2)	−0.4	MPa
25	τ	$\psi_{D2A} = \psi_{A2D} \times \tau$, ψ_{D2A} is the SWP threshold for microbial resuscitation	(0.1, 0.95)	0.25	—
26	ω	Exponential in SWP function for microbial dormancy or resuscitation	(1, 6)	4	—

[DOC]: dissolved organic carbon content (mg C g^{-1} soil); [MOC]: mineral-associated organic carbon content (mg C g^{-1} soil).

%Sand: percentage of sand in soil.

Supplementary Table 9. The minimized objective functions of 48 cases of the short-term glucose (ST_G), short-term cellulose (ST_C) and long-term cellulose (LT_C) datasets as calibrated against CO_2 and MBC by MEND model. See Methods for detail of relevant calculations.

Location	Ecosystem type	Substrate treatment	ST_G		ST_C		LT_C	
			CO_2	MBC	CO_2	MBC	CO_2	MBC
Iowa	Forest	Control	0.23	0.05	0.28	0.12	0.22	0.15
		C added	0.43	0.21	0.17	0.24	0.33	0.22
	Grassland	Control	0.27	0.06	0.22	0.04	0.33	0.17
		C added	0.36	0.09	0.28	0.03	0.39	0.14
Missouri	Forest	Control	0.32	0.11	0.32	0.12	0.35	0.16
		C added	0.35	0.16	0.22	0.04	0.26	0.18
	Grassland	Control	0.23	0.11	0.15	0.14	0.32	0.31
		C added	0.32	0.05	0.21	0.14	0.20	0.17
Ohio	Forest	Control	0.16	0.15	0.18	0.35	0.18	0.33
		C added	0.35	0.19	0.38	0.03	0.39	0.27
	Grassland	Control	0.10	0.03	0.21	0.05	0.26	0.08
		C added	0.29	0.06	0.22	0.29	0.24	0.28
Tennessee	Forest	Control	0.14	0.01	0.23	0.06	0.39	0.35
		C added	0.48	0.14	0.40	0.09	0.34	0.12
	Grassland	Control	0.13	0.01	0.14	0.07	0.15	0.10
		C added	0.34	0.12	0.04	0.19	0.11	0.22

Supplementary Table 10. Description of 149 field warming studies collected in our meta-analysis. Information on experimental duration being reported (Years), warming magnitude (ΔT), number of replicates (N.rep), carbon stocks (kg C m-^{2}) in control plots (C.control) and warmed plots (C.warmed), plot coordinates (Lat:latitude, Long: longtitude), mean annual temperature (MAT), mean annual precipitation (MAP), soil acidity (pH), experimental duration categorized in our meta-analysis (Duration), and data source.

ID	Years	ΔT	N.rep	C.control	C.warmed	Lat	Long	MAT	MAP	pH	Duration	Source
**1**	10.3	0.5	5	3.2	4.35	63.88	-145.74	-3.2	298	6.6	>10 yr	Crowther
**2**	5.0	4.6	4	3.62	3.03	46.64	-88.48	4.4	824	5.3	1-10 yr	Crowther
**3**	5.0	4.6	4	5.07	2.76	46.64	-88.48	4.4	824	5.3	1-10 yr	Crowther
**4**	4.0	1.0	5	5.86	5.53	78.17	16.10	-5.7	226	6	1-10 yr	Crowther
**5**	4.0	1.0	5	7.39	6.74	78.17	16.10	-5.7	226	6	1-10 yr	Crowther
**6**	15.0	0.2	3	11.99	11.09	53.06	-3.47	7.1	1215	5.2	>10 yr	Crowther
**7**	0.7	2.3	4	10.18	9.91	39.13	115.67	12.7	543	7	≤1 yr	Crowther
**8**	5.0	4.0	6	11.96	7.79	42.53	-72.19	6.8	1142	4.9	1-10 yr	Crowther
**9**	25.0	2.0	5	1.7	1.67	38.96	-106.99	0.5	519	5.8	> 10 yr	Crowther
**10**	14.0	0.4	3	0.53	0.52	46.86	19.42	10.9	536	7.1	> 10 yr	Crowther
**11**	6.0	0.9	6	1.01	0.81	55.88	11.97	8.2	603	5.5	1-10 yr	Crowther
**12**	2.0	1.0	3	4.48	4.39	64.03	18.93	1	609	4.6	1-10 yr	Crowther
**13**	2.0	1.8	6	1.4	1.31	37.41	-122.23	13.7	635	6.2	1-10 yr	Crowther
**14**	2.0	1.8	6	1.55	1.56	37.41	-122.23	13.7	635	6.2	1-10 yr	Crowther
**15**	5.0	2.6	5	2.8	2.91	36.01	-84.26	13.9	1347	5.6	1-10 yr	Crowther
**16**	5.0	2.6	5	2.73	3.09	36.01	-84.26	13.9	1347	5.6	1-10 yr	Crowther
**17**	10.0	1.5	6	2.74	2.18	34.98	-97.52	16.3	906	6.7	1-10 yr	Crowther
**18**	10.0	1.5	6	2.53	2.37	34.98	-97.52	16.3	906	6.7	1-10 yr	Crowther
**19**	3.0	1.8	6	2.03	1.95	44.75	123.75	5.2	436	7.9	1-10 yr	Crowther
**20**	4.0	3.0	3	3.69	4.73	36.03	-79.17	14.4	1161	4.9	1-10 yr	Crowther
**21**	4.0	5.0	3	3.69	4.26	36.03	-79.17	14.4	1161	4.9	1-10 yr	Crowther
**22**	4.0	1.0	3	4.74	4.76	39.08	-96.58	12	872	6.4	1-10 yr	Crowther
**23**	3.0	2.1	1	1.24	1.29	33.88	-83.35	16.5	1230	4.6	1-10 yr	Crowther
**24**	4.0	4.3	1	1.32	1.01	33.88	-83.35	16.5	1230	4.6	1-10 yr	Crowther
**25**	14.0	1.5	6	5.12	5.05	68.35	18.82	-0.1	390	5.1	>10 yr	Crowther

**26**	6.0	2.8	5	1.75	2	41.20	-104.89	7	384	7.4	1-10 yr	Crowther
**27**	4.5	0.9	3	2.44	2.48	41.30	1.82	15.5	632	6.8	1-10 yr	Crowther
**28**	3.0	2.0	4	1.38	1.55	48.72	9.19	8.9	729	6.3	1-10 yr	Crowther
**29**	3.0	2.0	4	1.33	1.53	48.72	9.19	8.9	729	6.3	1-10 yr	Crowther
**30**	3.0	2.0	4	1.25	1.53	48.72	9.19	8.9	729	6.3	1-10 yr	Crowther
**31**	3.0	2.0	4	1.4	1.55	48.72	9.19	8.9	729	6.3	1-10 yr	Crowther
**32**	3.0	2.0	4	1.35	1.44	48.72	9.19	8.9	729	6.3	1-10 yr	Crowther
**33**	0.4	2.5	3	1.36	1.39	46.68	-92.52	3.8	761	5.5	≤1 yr	Crowther
**34**	0.4	2.5	3	1.96	2.07	46.68	-92.52	3.8	761	5.5	≤1 yr	Crowther
**35**	0.4	2.5	3	1.49	1.38	46.68	-92.52	3.8	761	5.5	≤1 yr	Crowther
**36**	0.4	2.5	3	1.41	1.41	46.68	-92.52	3.8	761	5.5	≤1 yr	Crowther
**37**	0.4	2.5	3	1.48	1.54	46.68	-92.52	3.8	761	5.5	≤1 yr	Crowther
**38**	0.4	2.5	3	1.35	1.39	46.68	-92.52	3.8	761	5.5	≤1 yr	Crowther
**39**	2.2	2.8	5	4.26	3.86	44.03	-123.18	11.4	1194	5.3	1-10 yr	Crowther
**40**	2.2	2.8	5	4.27	4.1	44.03	-123.18	11.4	1194	5.3	1-10 yr	Crowther
**41**	2.2	3.0	5	3.18	3.3	42.28	-124.64	11.4	1364	5.5	1-10 yr	Crowther
**42**	1.8	2.9	5	3.64	4.08	46.90	-122.73	10.1	1199	5.3	1-10 yr	Crowther
**43**	2.2	3.0	5	3.56	3.44	42.28	-124.64	11.4	1364	5.5	1-10 yr	Crowther
**44**	1.8	2.9	5	4.13	3.9	46.90	-122.73	10.1	1199	5.3	1-10 yr	Crowther
**45**	4.0	0.9	3	4.76	3.88	56.23	10.57	7.4	592	5.3	1-10 yr	Crowther
**46**	20.0	0.5	4	2.46	2.11	68.63	-149.57	-11.2	237	6	> 10 yr	Crowther
**47**	0.8	4.8	4	7.64	6.35	43.95	-71.72	5.4	1082	5	≤1 yr	Crowther
**48**	9.0	2.0	4	0.21	0.16	76.53	-68.42	-11.3	112	6.9	1-10 yr	Crowther
**49**	9.0	2.0	4	2.14	2	76.53	-68.42	-11.3	112	6.9	1-10 yr	Crowther
**50**	10.8	2.7	6	2.59	2.84	68.33	20.85	-0.7	299	7.1	>10 yr	van Gestel
**51**	8.6	2.7	6	2.37	2.62	68.33	20.85	-0.7	299	7.1	1-10 yr	van Gestel
**52**	6.0	1.7	6	2.57	2.7	68.33	20.85	-0.7	299	7.1	1-10 yr	van Gestel
**53**	6.0	1.7	6	2.37	2.55	68.33	20.85	-0.7	299	7.1	1-10 yr	van Gestel
**54**	7.5	2.0	6	4.04	4.24	68.33	20.85	-0.7	299	7.1	1-10 yr	van Gestel
**55**	13.0	2.8	6	5.75	5.95	68.33	20.85	-0.7	299	7.1	>10 yr	van Gestel

**56**	5.0	3.2	6	0.19	0.2	68.30	20.80	-4.8	500	5.2	1-10 yr	van Gestel
**57**	6.0	3.1	6	0.17	0.16	68.30	20.80	-4.8	500	5.2	1-10 yr	van Gestel
**58**	5.0	1.8	6	0.19	0.23	68.30	20.80	-4.8	500	5.2	1-10 yr	van Gestel
**59**	6.0	1.5	6	0.17	0.26	68.30	20.80	-4.8	500	5.2	1-10 yr	van Gestel
**60**	9.0	2.3	6	3.36	3.61	68.35	20.82	-0.7	299	4.2	1-10 yr	van Gestel
**61**	9.0	2.2	6	3.39	3.77	68.35	20.82	-0.7	299	4.2	1-10 yr	van Gestel
**62**	2.2	1.3	5	0.39	0.44	68.35	18.82	-0.7	304	5.22	1-10 yr	van Gestel
**63**	2.2	1.3	5	3.83	4.19	68.35	18.82	-0.7	304	3.86	1-10 yr	van Gestel
**64**	4.0	2.1	5	3.13	3.27	24.53	101.00	11.3	1778	4.5	1-10 yr	van Gestel
**65**	3.3	1.3	10	1.09	1.31	-64.78	-64.00	-1.7	750	6	1-10 yr	van Gestel
**66**	3.3	1.3	10	0.7	1.05	-64.78	-64.00	-1.7	750	6	1-10 yr	van Gestel
**67**	3.8	2.9	5	0.25	0.34	40.03	-3.32	15	349	7	1-10 yr	van Gestel
**68**	3.8	2.9	5	0.24	0.31	40.03	-3.32	15	349	7	1-10 yr	van Gestel
**69**	3.8	2.9	5	0.1	0.19	40.03	-3.32	15	349	7	1-10 yr	van Gestel
**70**	3.8	2.9	5	0.14	0.13	40.03	-3.32	15	349	7	1-10 yr	van Gestel
**71**	2.9	3.1	3	6.63	6.71	42.38	-71.87	9.5	1194	5.5	1-10 yr	van Gestel
**72**	2.9	3.1	3	7.06	7.04	42.38	-71.87	9.5	1194	5.5	1-10 yr	van Gestel
**73**	2.9	3.1	3	6.54	6.77	42.38	-71.87	9.5	1194	5.5	1-10 yr	van Gestel
**74**	2.9	0.8	3	6.63	6.61	42.38	-71.87	9.5	1194	5.5	1-10 yr	van Gestel
**75**	2.9	0.8	3	7.06	7.5	42.38	-71.87	9.5	1194	5.5	1-10 yr	van Gestel
**76**	2.9	0.8	3	6.54	6.56	42.38	-71.87	9.5	1194	5.5	1-10 yr	van Gestel
**77**	2.9	2.3	3	6.63	6.97	42.38	-71.87	9.5	1194	5.5	1-10 yr	van Gestel
**78**	2.9	2.3	3	7.06	6.95	42.38	-71.87	9.5	1194	5.5	1-10 yr	van Gestel
**79**	2.9	2.3	3	6.54	6.64	42.38	-71.87	9.5	1194	5.5	1-10 yr	van Gestel
**80**	4.4	0.8	6	4.45	3.9	55.88	11.97	8	613	4.5	1-10 yr	van Gestel
**81**	4.4	0.8	6	4.98	4.79	55.88	11.97	8	613	4.5	1-10 yr	van Gestel
**82**	4.4	0.8	6	4.57	3.95	55.88	11.97	8	613	4.5	1-10 yr	van Gestel
**83**	9.0	2.0	3	2	3.71	78.90	-75.92	-14.6	150	6.6	1-10 yr	van Gestel
**84**	9.0	2.0	4	6.23	4.52	78.90	-75.92	-14.6	150	6.6	1-10 yr	van Gestel
**85**	9.0	2.0	3	5.9	5.44	78.90	-75.92	-14.6	150	6.6	1-10 yr	van Gestel

**86**	2.0	3.0	5	4.38	4.53	-43.03	171.75	10	1300	5.4	1-10 yr	van Gestel
**87**	2.0	3.0	5	4.71	4.63	-43.03	171.75	10	1300	5.4	1-10 yr	van Gestel
**88**	1.0	2.5	3	1.31	1.31	45.58	-93.17	6.8	799	6	≤1 yr	van Gestel
**89**	1.0	2.5	3	1.51	1.24	45.58	-93.17	6.8	799	6	≤1 yr	van Gestel
**90**	1.0	2.5	3	1.93	1.33	45.58	-93.17	6.8	799	6	≤1 yr	van Gestel
**91**	1.0	2.5	3	1.53	1.34	45.58	-93.17	6.8	799	6	≤1 yr	van Gestel
**92**	1.0	2.5	3	2.05	1.98	45.58	-93.17	6.8	799	6	≤1 yr	van Gestel
**93**	1.0	2.5	3	1.13	1.95	45.58	-93.17	6.8	799	6	≤1 yr	van Gestel
**94**	1.0	2.5	3	1.38	1.36	45.58	-93.17	6.8	799	6	≤1 yr	van Gestel
**95**	1.0	2.5	3	1.43	1.59	45.58	-93.17	6.8	799	6	≤1 yr	van Gestel
**96**	1.3	1.4	4	12.05	11.62	30.50	91.06	1.3	477	6.35	1-10 yr	van Gestel
**97**	1.3	1.3	4	5.13	5.13	30.50	91.06	1.3	477	6.35	1-10 yr	van Gestel
**98**	1.3	1.0	4	6.47	5.87	30.50	91.06	1.3	477	6.35	1-10 yr	van Gestel
**99**	2.6	1.0	6	5.73	5.55	63.88	-149.23	-1	378	4.823	1-10 yr	van Gestel
**100**	2.2	1.3	5	4.08	4.19	62.30	9.62	1.15	473	6.18	1-10 yr	van Gestel
**101**	5.3	1.2	6	3.28	3.15	42.03	116.28	2.1	382.3	6.84	1-10 yr	van Gestel
**102**	5.3	1.4	6	2.45	2.47	42.03	116.28	2.1	382.3	6.84	1-10 yr	van Gestel
**103**	3.9	1.8	6	4.71	4.9	42.03	116.28	2.1	382.3	6.84	1-10 yr	van Gestel
**104**	14.4	5.0	2	1.2	1.1	64.10	19.45	2	600	4.4	>10 yr	van Gestel
**105**	2.0	1.3	4	6.21	5.45	37.60	101.30	-1.7	561	7.3	1-10 yr	van Gestel
**106**	7.0	4.0	3	5.1	4.17	42.70	141.60	6.3	1450	5.1	1-10 yr	van Gestel
**107**	7.6	1.8	6	1.96	1.87	37.40	-122.23	14	652	6.8	1-10 yr	van Gestel
**108**	7.6	1.8	6	1.83	1.85	37.40	-122.23	14	652	6.8	1-10 yr	van Gestel
**109**	7.6	1.8	6	1.77	1.83	37.40	-122.23	14	652	6.8	1-10 yr	van Gestel
**110**	7.6	1.8	6	1.82	1.88	37.40	-122.23	14	652	6.8	1-10 yr	van Gestel
**111**	7.6	1.8	6	1.78	1.72	37.40	-122.23	14	652	6.8	1-10 yr	van Gestel
**112**	7.6	1.8	6	1.93	1.88	37.40	-122.23	14	652	6.8	1-10 yr	van Gestel
**113**	2.2	0.1	5	1.33	1.34	69.75	23.98	-1.5	354	4.07	1-10 yr	van Gestel
**114**	2.2	1.2	5	1.28	1.23	69.75	23.98	-1.5	354	4.03	1-10 yr	van Gestel
**115**	10.6	2.0	4	7.45	10.77	68.35	18.50	-2	848	3.7	>10 yr	van Gestel

**116**	10.6	2.0	4	8.33	7.62	68.35	18.50	-2	848	4.7	>10 yr	van Gestel
**117**	4.0	0.9	4	3.46	5.63	31.58	102.58	8.9	790	6.19	1-10 yr	van Gestel
**118**	10.0	1.0	4	6.38	6.13	31.44	101.17	-3	450	7.06	1-10 yr	van Gestel
**119**	13.0	1.3	3	3.54	3.83	52.40	5.90	8.3	1042	3.8	> 10 yr	van Gestel
**120**	3.5	2.2	3	3.87	3.76	35.90	-84.34	14.2	1322	5.8	1-10 yr	van Gestel
**121**	3.5	1.4	3	3.8	3.86	35.90	-84.34	14.2	1322	5.8	1-10 yr	van Gestel
**122**	3.5	1.4	3	3.82	4	35.90	-84.34	14.2	1322	5.8	1-10 yr	van Gestel
**123**	3.5	2.2	3	3.89	4.13	35.90	-84.34	14.2	1322	5.8	1-10 yr	van Gestel
**124**	3.0	1.6	3	2.54	2.46	32.20	121.12	17.7	1044.7	6.1	1-10 yr	van Gestel
**125**	2.0	2.3	5	0.65	0.65	34.82	92.92	-3.8	291	8.35	1-10 yr	van Gestel
**126**	3.0	2.0	3	2.15	2.22	34.85	92.93	-3.8	383	7.7	1-10 yr	van Gestel
**127**	11.0	0.2	3	3.17	3.28	40.60	8.15	16.8	610	7.3	>10 yr	van Gestel
**128**	1.3	2.3	9	12.66	13.91	67.90	74.90	-8.8	370	5.9	1-10 yr	van Gestel
**129**	3.7	2.9	5	8.33	8.48	31.70	103.90	8.9	920	5.55	1-10 yr	van Gestel
**130**	3.9	0.7	6	8	9.02	33.00	104.00	2.85	813	5.8	1-10 yr	van Gestel
**131**	3.9	0.7	6	7.47	7.75	33.00	104.00	2.85	813	5.8	1-10 yr	van Gestel
**132**	3.3	2.9	5	8.97	9.16	31.70	103.90	8.9	920	5.55	1-10 yr	van Gestel
**133**	5.9	1.8	3	3.03	2.9	-42.70	147.27	11.6	560	5.86	1-10 yr	van Gestel
**134**	5.9	1.8	3	3.43	3.48	-42.70	147.27	11.6	560	5.86	1-10 yr	van Gestel
**135**	5.9	1.8	3	3.68	4.31	-42.70	147.27	11.6	560	5.86	1-10 yr	van Gestel
**136**	5.9	1.8	3	3.59	3.3	-42.70	147.27	11.6	560	5.86	1-10 yr	van Gestel
**137**	3.0	2.0	5	0.53	0.5	38.67	-109.42	12.2	236	7.8	1-10 yr	van Gestel
**138**	1.5	2.0	5	0.3	0.28	38.67	-109.42	12.2	236	7.8	1-10 yr	van Gestel
**139**	1.5	2.0	5	0.3	0.3	38.67	-109.42	12.2	236	7.8	1-10 yr	van Gestel
**140**	2.1	2.0	8	6.16	5.69	39.30	-111.50	1.7	902	6.4	1-10 yr	van Gestel
**141**	2.1	2.0	8	5.09	5.05	39.30	-111.50	1.7	902	6.4	1-10 yr	van Gestel
**142**	2.1	2.0	8	4.76	5.12	39.30	-111.50	1.7	902	6.4	1-10 yr	van Gestel
**143**	2.1	2.0	8	5.78	6.71	39.30	-111.50	1.7	902	6.4	1-10 yr	van Gestel
**144**	7.0	1.2	4	2.87	2.87	30.30	91.00	1.9	474.9	6.5	1-10 yr	Fu et al. 2019
**145**	7.0	1.3	4	3.11	3.43	30.30	91.00	1.9	474.9	6.1	1-10 yr	Fu et al. 2019

**146**	7.0	1.2	4	6.52	6.9	30.30	91.00	1.9	474.9	5.6	1-10 yr	Fu et al. 2019
**147**	5.0	1.1	2	0.69	0.6	42.00	116.28	2.1	385	7.2	1-10 yr	Zhang et al. 2017
**148**	3.0	3.0	3	10.8	10.81	36.00	137.40	6.4	2075		1-10 yr	Noh et al. 2017
**149**	2.5	2.5	3	0.44	0.55	31.70	103.88	9.3	825.2		1-10 yr	Liu et al. 2018

Supplementary Figures

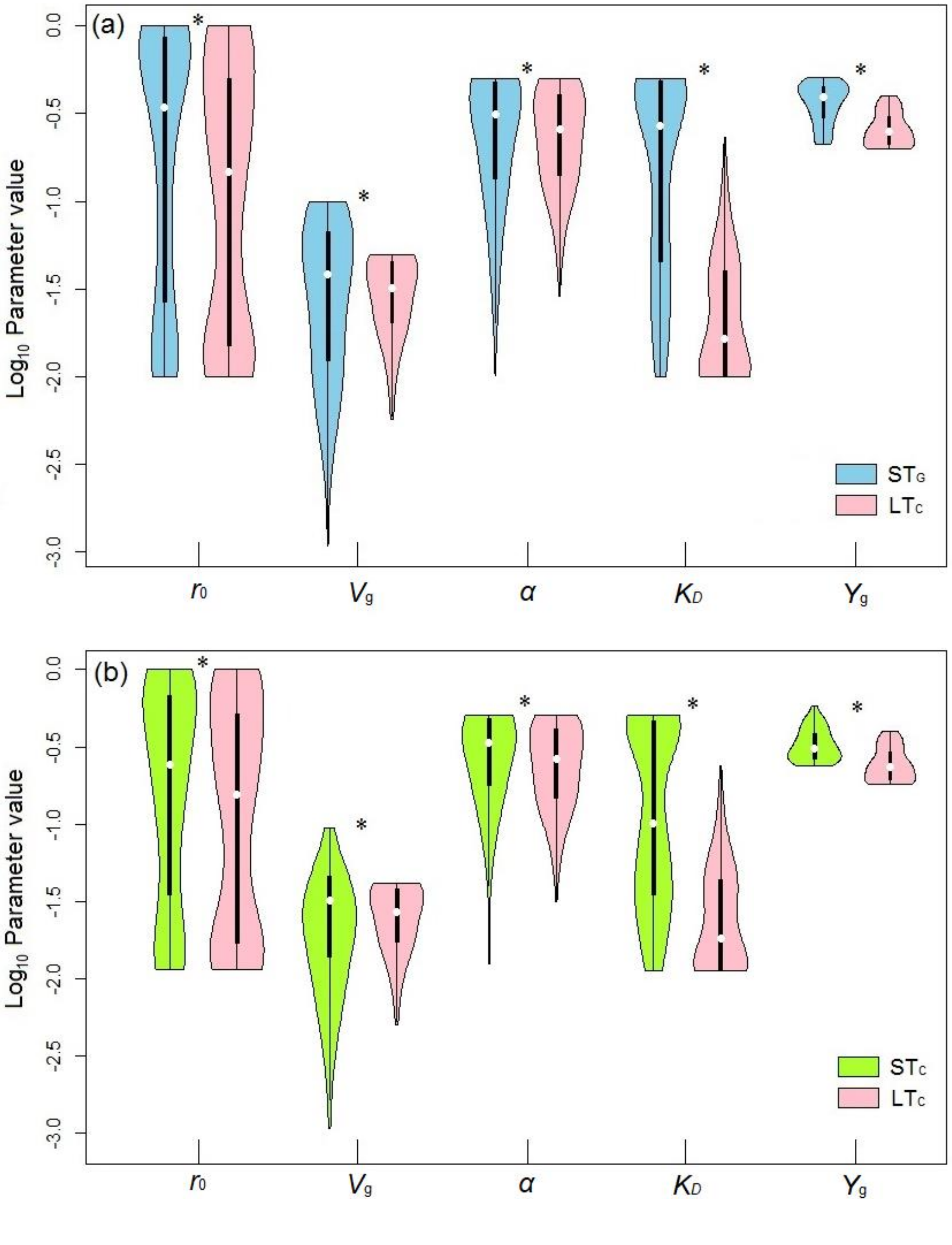

(a)
Log10 Parameter value
0.0
-0.5
-1.0
-1.5
-2.0
-2.5
-3.0
*
r0
Vg
α
KD
Yg
STG
LTC
(b)
Log10 Parameter value
0.0
-0.5
-1.0
-1.5
-2.0
-2.5
-3.0
*
r0
Vg
α
KD
Yg
STC
LTC

Supplementary Figure 1. Uncertainty distributions of five microbial parameters (*r_0, V_g, α, K_D, Y_g*) derived for (a) the short-term glucose (ST_G) and long-term cellulose (LT_C) datasets, and (b) for the short-term cellulose (ST_C) and long-term cellulose (LT_C) datasets. * denotes significantly different distribution tested by Kruskal-Wallis (KW) method at α=0.05. Violin plots show medians (white dot), 1st and 3rd quartiles (black bar, interquartile range or IQR), and upper and lower extremes (line). The whiskers were determined as equal to or less than 1.5 times IQR against 1st and 3rd quartiles, respectively.

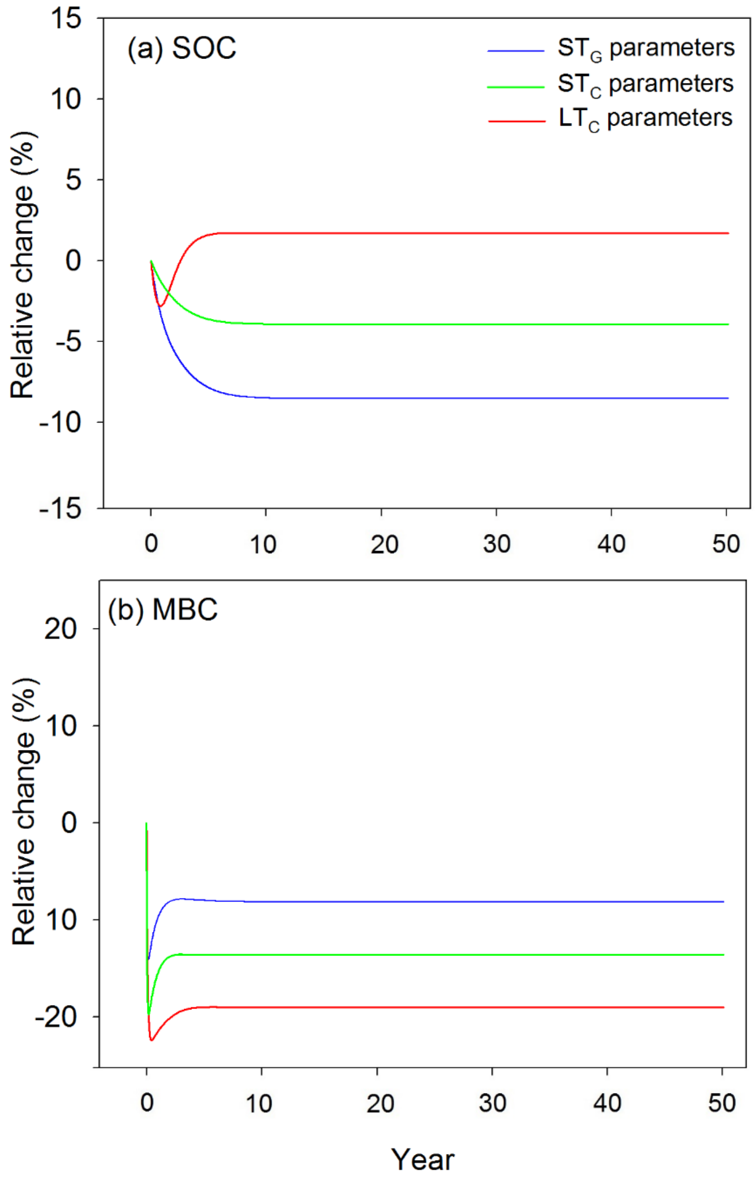

Supplementary Figure 2. Five-decade long dynamics of modeled relative changes (%) in (a) SOC and (b) MBC with 5 °C warming by implementing the best-fit short-term glucose (ST_G), short-term cellulose (ST_C) and long-term cellulose (LT_C) datasets derived parameters.

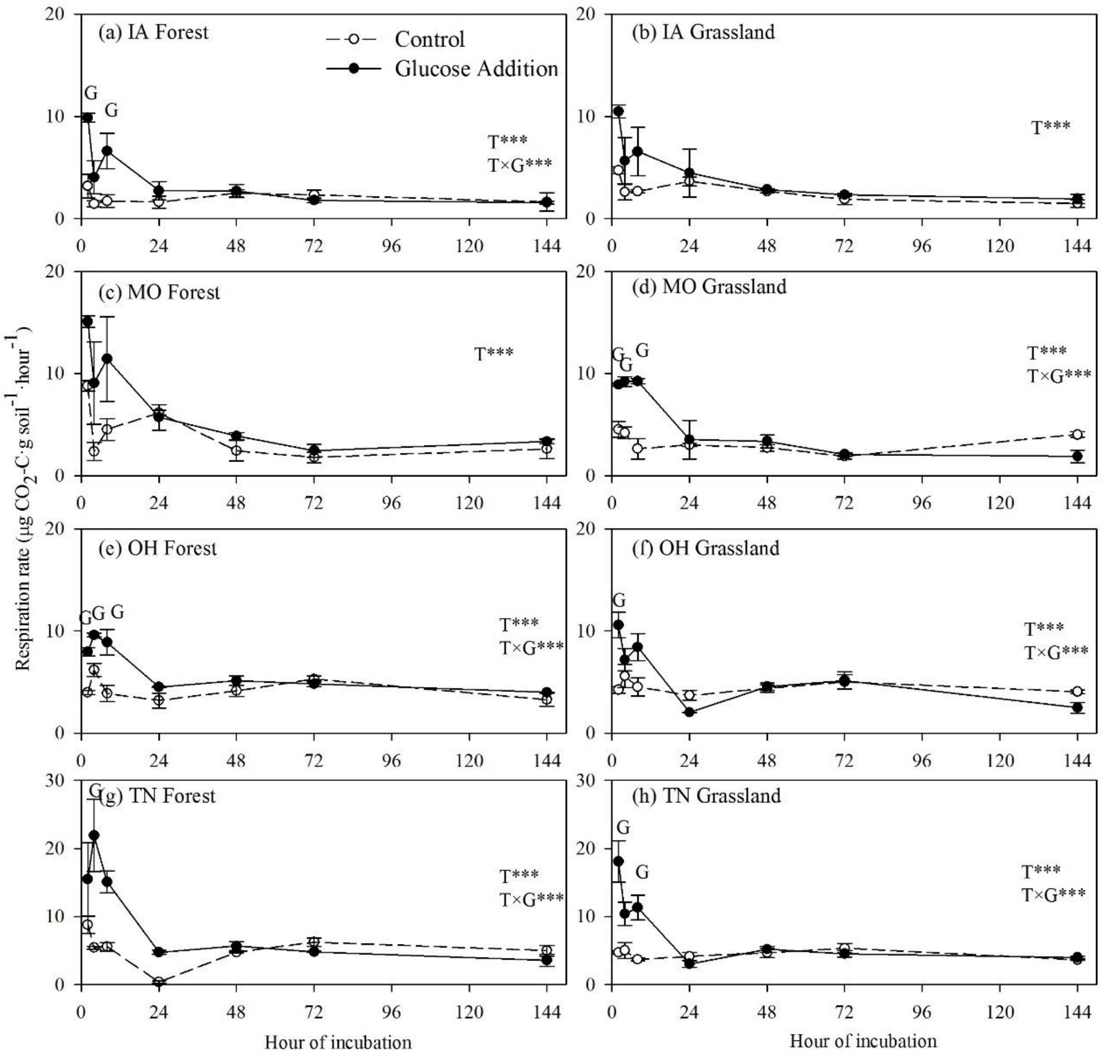

Supplementary Figure 3. Mean ± standard error of respiration rate (µg CO_2-C g^{-1} soil $hour^{-1}$) from short-term incubation. T denotes significant time effect, G denotes significant glucose addition effect on respiration rate in each collection. * denotes $p<0.05$, ** denotes $p<0.01$, *** denotes $p<0.001$. N=3 independent samples for each collection. Two-sided p values were obtained.

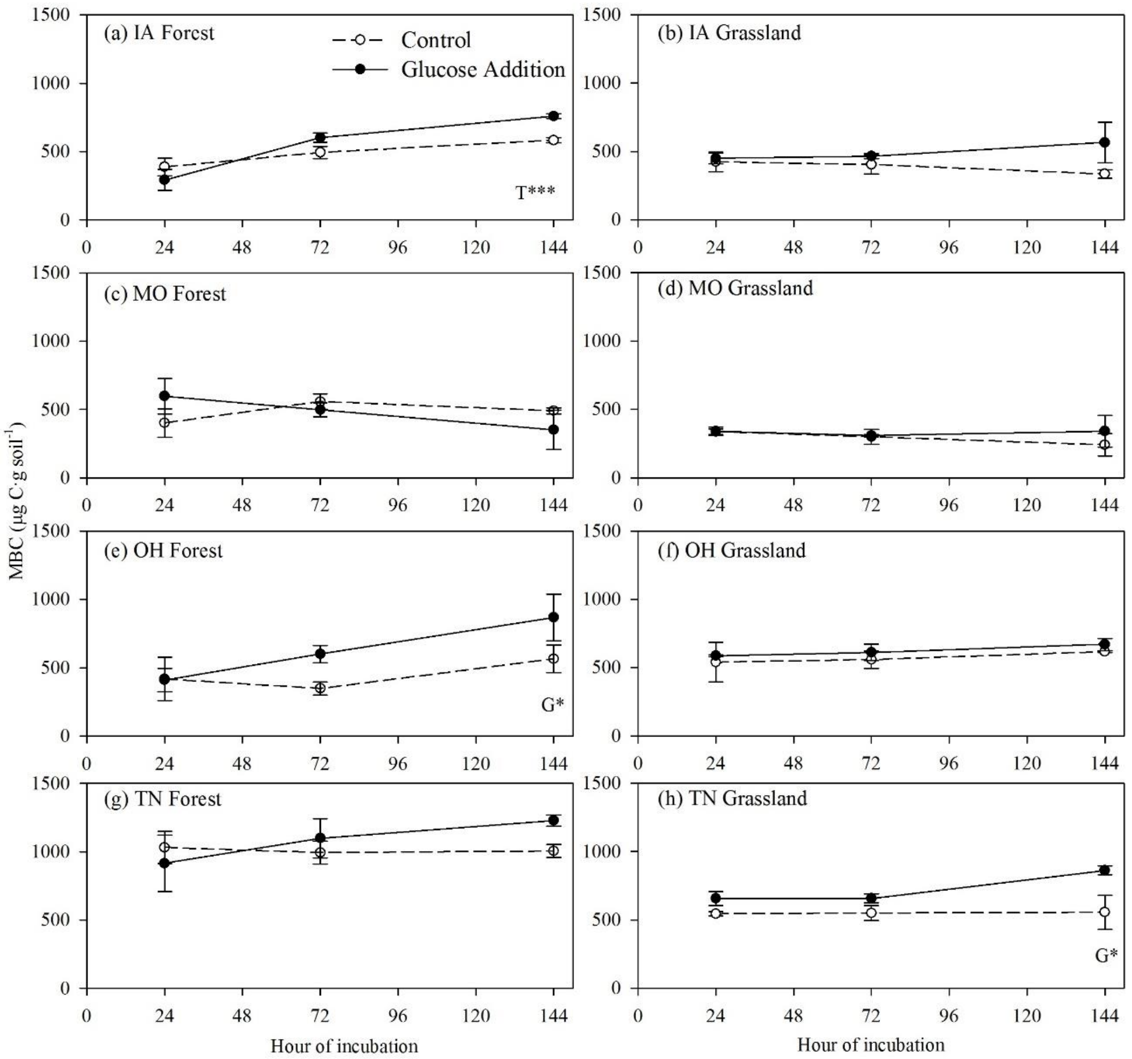

Supplementary Figure 4. Mean ± standard error of microbial biomass carbon (MBC, μg C g^{-1} soil) from short-term incubation. T denotes significant time effect, G denotes significant cellulose addition effect. * denotes $p<0.05$, ** denotes $p<0.01$, *** denotes $p<0.001$. N=3 independent samples for each collection. Two-sided p values were obtained.

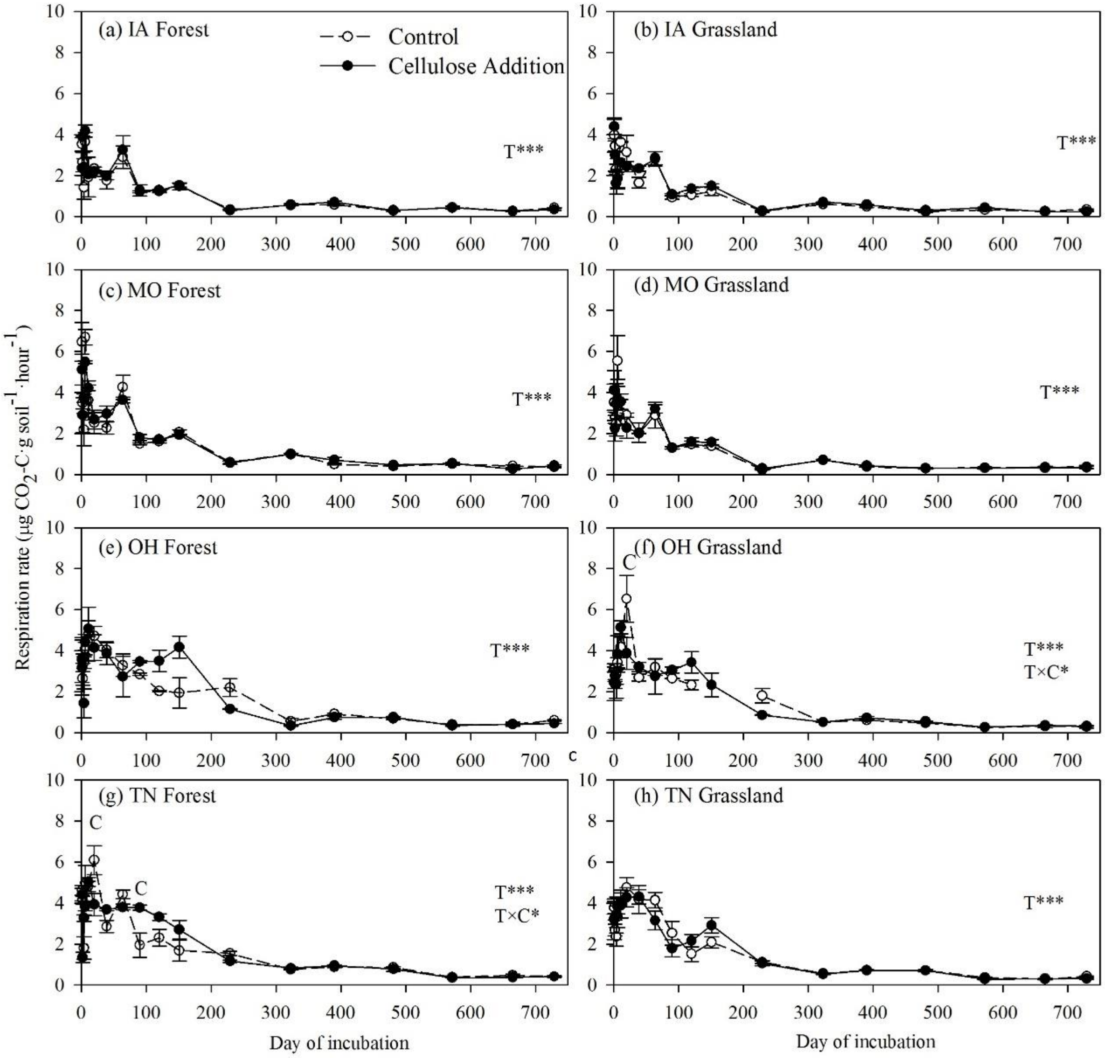

Supplementary Figure 5. Mean ± standard error of respiration rate (μg CO_2-C g^{-1} soil $hour^{-1}$) from long-term incubation. T denotes significant time effect, C denotes significant cellulose addition effect. * denotes $p<0.05$, ** denotes $p<0.01$, *** denotes $p<0.001$. N=3 independent samples for each collection. Two-sided p values were obtained.

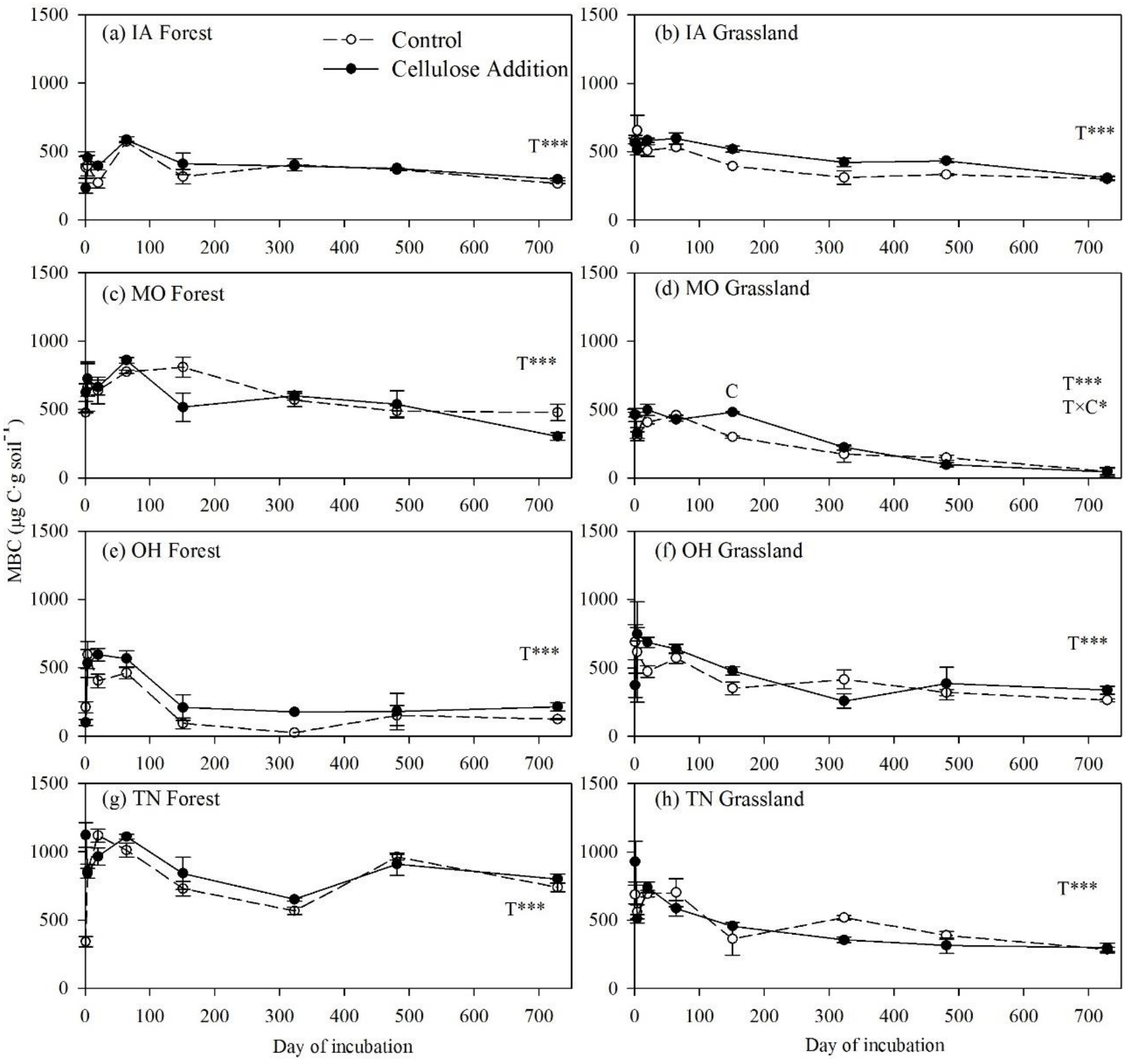

Supplementary Figure 6. Mean ± standard error of microbial biomass carbon (MBC, μg C g^{-1} soil) from long-term incubation. T denotes significant time effect, C denotes significant cellulose addition effect. * denotes p<0.05, ** denotes p<0.01, *** denotes p<0.001. N=3 independent samples for each collection. Two-sided p values were obtained.

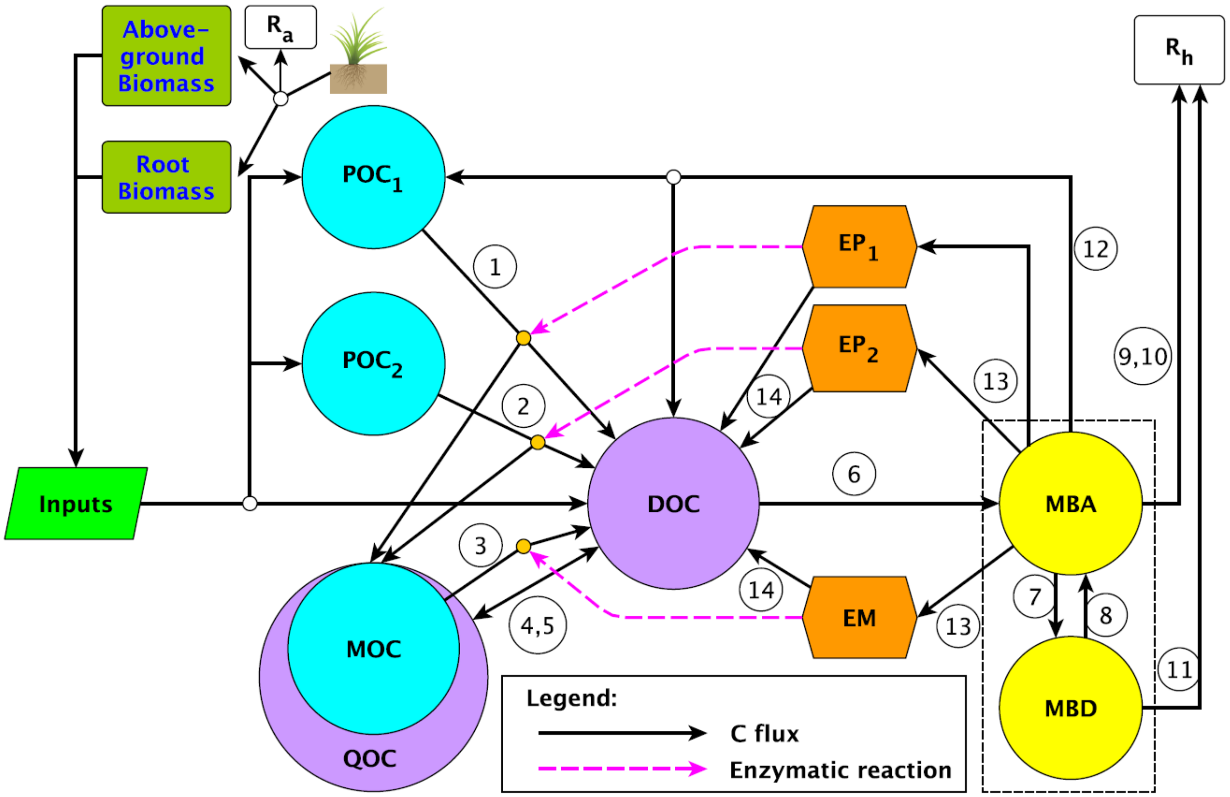

Supplementary Figure 7. Diagram of the Microbial-ENzyme Decomposition (MEND) model[9, 10, 14]. Soil organic carbon pools include: (1) particulate organic C (POC), which can be further divided into two components POC_1 (denoted by state variable P_1 in governing equations) degraded by oxidative enzymes EP1 and POC_2 (P_2 degraded by hydrolytic enzymes EP2; (2) mineral-associated organic C (MOC, *M*) degraded by enzymes EM; (3) dissolved organic C (DOC, *D*); (4) adsorbed DOC (QOC, *Q*): active MOC that adsorbs and desorbs DOC; (5) active microbial biomass (MBA, *BA*) and dormant microbial biomass (MBD, *BD*); (6) enzyme pools EP_1, EP_2 and *EM*. External litter inputs (Inputs) can be separated into I_{P1}, I_{P2} and I_D denoting inputs to the pools of POC_1, POC_2, and DOC, respectively. R_a and R_h represent autotrophic and heterotrophic respiration, respectively.

Supplementary References

1. Kluber A*, et al.* Soil Respiration and Microbial Biomass from Soil Incubations with 13C Labeled Additions. *Oak Ridge National Laboratory, TES SFA, US Department of Energy, Oak Ridge, Tennessee, USA* [*https://tes-sfaornlgov/node/80*](https://tes-sfaornlgov/node/80), (2020).

2. Soil Survey Staff U. Web soil survey. *USDA Natural Resources Conservation Service*, (2013).

3. Gee GW, Or D. 2.4 Particle-size analysis. *Methods of soil analysis Part* **4**, 255-293 (2002).

4. Thomas GW. Soil pH and soil acidity. *Methods of soil analysis: part 3 chemical methods* **5**, 475-490 (1996).

5. Nelson DW, Sommers LE. Total carbon, organic carbon, and organic matter. *Methods of soil analysis part 3—chemical methods*, 961-1010 (1996).

6. Vance ED, Brookes PC, Jenkinson DS. An extraction method for measuring soil microbial biomass C. *Soil biology and Biochemistry* **19**, 703-707 (1987).

7. Beck T*, et al.* An inter-laboratory comparison of ten different ways of measuring soil microbial biomass C. *Soil Biology and Biochemistry* **29**, 1023-1032 (1997).

8. Jagadamma S, Mayes MA, Steinweg JM, Schaeffer SM. Substrate quality alters the microbial mineralization of added substrate and soil organic carbon. *Biogeosciences* **11**, 4665-4678 (2014).

9. Wang GS, Post WM, Mayes MA. Development of microbial-enzyme-mediated decomposition model parameters through steady-state and dynamic analyses. *Ecological Applications* **23**, 255-272 (2013).

10. Wang GS*, et al.* Soil moisture drives microbial controls on carbon decomposition in two subtropical forests. *Soil Biol Biochem* **130**, 185-194 (2019).

11. van Gestel N*, et al.* Predicting soil carbon loss with warming. *Nature* **554**, E4-E5 (2018).

12. Rinnan R, Michelsen A, Jonasson S. Effects of litter addition and warming on soil carbon, nutrient pools and microbial communities in a subarctic heath ecosystem. *Applied Soil Ecology* **39**, 271-281 (2008).

13. Allison SD, Wallenstein MD, Bradford MA. Soil-carbon response to warming dependent on microbial physiology. *Nature Geoscience* **3**, 336-340 (2010).

14. Wang GS*, et al.* Microbial dormancy improves development and experimental validation of ecosystem model. *Isme J* **9**, 226-237 (2015).
