# Supplementary material for: Multi-year incubation experiments boost confidence in model projections of long-term soil carbon dynamics
Source: Nat Commun. 2020 Nov 17;11:5864. doi: 10.1038/s41467-020-19428-y (PMC7672078; doi:10.1038/s41467-020-19428-y)
Supplement: Supplementary file 2 — Reporting Summary [file 41467_2020_19428_MOESM2_ESM.pdf]

nature research

Corresponding author(s): Jianwei Li

Last updated by author(s): Oct 9, 2020

Reporting Summary

Nature Research wishes to improve the reproducibility of the work that we publish. This form provides structure for consistency and transparency in reporting. For further information on Nature Research policies, see our Editorial Policies and the Editorial Policy Checklist.

Statistics

For all statistical analyses, confirm that the following items are present in the figure legend, table legend, main text, or Methods section.

n/a	Confirmed	
☐	☒	The exact sample size (*n*) for each experimental group/condition, given as a discrete number and unit of measurement
☐	☒	A statement on whether measurements were taken from distinct samples or whether the same sample was measured repeatedly
☐	☒	The statistical test(s) used AND whether they are one- or two-sided<br>*Only common tests should be described solely by name; describe more complex techniques in the Methods section.*
☐	☒	A description of all covariates tested
☐	☒	A description of any assumptions or corrections, such as tests of normality and adjustment for multiple comparisons
☐	☒	A full description of the statistical parameters including central tendency (e.g. means) or other basic estimates (e.g. regression coefficient) AND variation (e.g. standard deviation) or associated estimates of uncertainty (e.g. confidence intervals)
☐	☒	For null hypothesis testing, the test statistic (e.g. F, t, r) with confidence intervals, effect sizes, degrees of freedom and P value noted<br>*Give P values as exact values whenever suitable.*
☐	☒	For Bayesian analysis, information on the choice of priors and Markov chain Monte Carlo settings
☐	☒	For hierarchical and complex designs, identification of the appropriate level for tests and full reporting of outcomes
☐	☒	Estimates of effect sizes (e.g. Cohen's d, Pearson's r), indicating how they were calculated

Our web collection on statistics for biologists contains articles on many of the points above.

Software and code

Policy information about availability of computer code

Data collection	GetData Graph Digitizer 2.2.6; The Shuffled Complex Evolution (SCE) algorithm used for model parameter calibration is embedded in the MEND model (https://github.com/wanggangsheng/MEND_mult.git)
Data analysis	The R software (version 3.4.0)

For manuscripts utilizing custom algorithms or software that are central to the research but not yet described in published literature, software must be made available to editors and reviewers. We strongly encourage code deposition in a community repository (e.g. GitHub). See the Nature Research guidelines for submitting code & software for further information.

Data

Policy information about availability of data

All manuscripts must include a data availability statement. This statement should provide the following information, where applicable:

- Accession codes, unique identifiers, or web links for publicly available datasets
- A list of figures that have associated raw data
- A description of any restrictions on data availability

Datasets used for the modeling study is available online as in Kluber et al.(https://tes-sfa.ornl.gov/node/80);

April 2020

Field-specific reporting

Please select the one below that is the best fit for your research. If you are not sure, read the appropriate sections before making your selection.

☐ Life sciences ☐ Behavioural & social sciences ☒ Ecological, evolutionary & environmental sciences

For a reference copy of the document with all sections, see nature.com/documents/nr-reporting-summary-flat.pdf

Ecological, evolutionary & environmental sciences study design

All studies must disclose on these points even when the disclosure is negative.

Study description	In this study, three incubation datasets derived from the same soils but of varying durations (6 days, 30 days and 729 days) were used to tune a soil microbial model and obtain best-fit microbial parameters. By implementing these parameters in the Microbial-ENzyme Decomposition (MEND) model, soil organic carbon (SOC) responses to warming were projected and compared with observations. The three sets of parameters derived from the two short- and one long-term datasets were implemented to project the SOC change in response to 5 °C warming over five decades. To validate the three sets of parameters, the model projections were compared with a meta-analysis of SOC responses to field warming manipulations of variable duration that consisted of 149 observations.
Research sample	The short- and long-term datasets used for model calibration were collected from a soil incubation study conducted at Oak Ridge National Laboratory from 2015 to 2017. (Kluber LA, et al. Soil Respiration and Microbial Biomass from Soil Incubations with 13C Labeled Additions. Oak Ridge National Laboratory, TES SFA, US Department of Energy, Oak Ridge, Tennessee, USA https://tes-sfa.ornl.gov/node/80)
Sampling strategy	The incubation dataset includes 48 cases (4 locations × 2 ecosystems × 2 substrate treatments × 3 durations) in total. The soil samples used in the incubation studies consist of 4 different soil types and 2 different ecosystems from the continental US. The heterotrophic respiration rate (CO2) and microbial biomass carbon (MBC) data were compiled for the data-model integration. For each case there are 10 (6 days and 30 days) or 18 (729 days) observation data points to calibrate five microbial parameters in the MEND model. The observation is sufficient to derive robust parameter estimates and represents different soils.
Data collection	The incubation dataset used for the model study was collected by LK, CS and MM at Oak Ridge National Laboratory using a modified Precon device coupled to a Thermo Fisher Delta V Advantage Isotope Ratio Mass Spectrometer (Thermo Fisher, Bremen, Germany), Shimadzu TOC-L analyzer and LECO Combustion Analyzer; All data were recorded in Excel file;<br><br>The meta-analysis was conducted by SJ at Tennessee State University using search engine (e.g., web of science), and GetData Graph Digitizer 2.2.6. All data were recorded in Excel file.
Timing and spatial scale	The model calibrations were conducted given the length of incubation datasets (6 days, 30 days, 90 days, 180 days, 360 days, 480 days and 729 days). The model projections on SOC responses to warming were conducted over five decades. Site level datasets were applied for model calibrations.
Data exclusions	There were no data exclusions because all incubation data were applied in the model study.
Reproducibility	The incubation datasets used in this study can be accessed via https://tes-sfa.ornl.gov/node/80. The parameter estimates derived from incubation datasets and the model projections based on these parameter estimates are provided in Supplementary file.
Randomization	Filed soil sampling used a random block design. During model calibration, parameter values were selected randomly.
Blinding	The blinding is not applicable because the model requires explicit time and location as well as treatment information.

Did the study involve field work? ☐ Yes ☒ No

Reporting for specific materials, systems and methods

We require information from authors about some types of materials, experimental systems and methods used in many studies. Here, indicate whether each material, system or method listed is relevant to your study. If you are not sure if a list item applies to your research, read the appropriate section before selecting a response.

Materials & experimental systems

n/a	Involved in the study
☒	☐ Antibodies
☒	☐ Eukaryotic cell lines
☒	☐ Palaeontology and archaeology
☒	☐ Animals and other organisms
☒	☐ Human research participants
☒	☐ Clinical data
☒	☐ Dual use research of concern

Methods

n/a	Involved in the study
☒	☐ ChIP-seq
☒	☐ Flow cytometry
☒	☐ MRI-based neuroimaging
